# Automated, Efficient, and Accelerated Knowledge Modeling of the Cognitive Neuroimaging Literature Using the ATHENA Toolkit

**DOI:** 10.3389/fnins.2019.00494

**Published:** 2019-05-15

**Authors:** Michael C. Riedel, Taylor Salo, Jason Hays, Matthew D. Turner, Matthew T. Sutherland, Jessica A. Turner, Angela R. Laird

**Affiliations:** ^1^Department of Physics, Florida International University, Miami, FL, United States; ^2^Department of Psychology, Florida International University, Miami, FL, United States; ^3^Psychology and Neuroscience, Georgia State University, Atlanta, GA, United States

**Keywords:** annotation, text-mining, neuroimaging, machine-learning, classification, ontology

## Abstract

Neuroimaging research is growing rapidly, providing expansive resources for synthesizing data. However, navigating these dense resources is complicated by the volume of research articles and variety of experimental designs implemented across studies. The advent of machine learning algorithms and text-mining techniques has advanced automated labeling of published articles in biomedical research to alleviate such obstacles. As of yet, a comprehensive examination of document features and classifier techniques for annotating neuroimaging articles has yet to be undertaken. Here, we evaluated which combination of corpus (abstract-only or full-article text), features (bag-of-words or Cognitive Atlas terms), and classifier (Bernoulli naïve Bayes, *k*-nearest neighbors, logistic regression, or support vector classifier) resulted in the highest predictive performance in annotating a selection of 2,633 manually annotated neuroimaging articles. We found that, when utilizing full article text, data-driven features derived from the text performed the best, whereas if article abstracts were used for annotation, features derived from the Cognitive Atlas performed better. Additionally, we observed that when features were derived from article text, anatomical terms appeared to be the most frequently utilized for classification purposes and that cognitive concepts can be identified based on similar representations of these anatomical terms. Optimizing parameters for the automated classification of neuroimaging articles may result in a larger proportion of the neuroimaging literature being annotated with labels supporting the meta-analysis of psychological constructs.

## Introduction

Neuroimaging research offers the potential to improve understanding of the neural mechanisms supporting a wide range of mental operations linked with mental health disorders and impacted by treatment interventions. These research endeavors are increasing in volume and scope, requiring “big data” methods to harness and translate this accumulated knowledge into improved cognitive models and ultimately intervention strategies. For example, a search of the National Center for Biotechnology Information PubMed engine^1^ identified over 121,000 publications from 2007 to 2012 matching the terms “fMRI” or “functional magnetic resonance imaging.” That number has risen to nearly 150,000 in the last 5 years, indicating that continued growth is to be expected. This body of literature represents a vast knowledge archive capturing a system-level perspective of functional brain organization. This includes a variety of motor (e.g., hand/body movements, speech), perceptual (e.g., visual, auditory), cognitive (e.g., memory, language, attention), affective (e.g., personality, emotion, mood), and interoceptive (e.g., hunger, thirst, micturition) systems. Capturing and discriminating the neurocognitive concepts across this plethora of information in an automated fashion for harvesting and data synthesis has yet to be sufficiently accomplished.

Biomedical text mining approaches have shown to be increasingly beneficial for extracting knowledge locked within text (Wang et al., 2007; Van Auken et al., 2012; Funk et al., 2014; Torii et al., 2014; Collier et al., 2015; Kim et al., 2015). Journal articles, patient electronic records, and social media posts may be mined to identify and predict relations among entities; for example, “*drug X causes adverse event Y.*” In various genomics or proteomics knowledge repositories, one focus has been to identify specific relationships between concepts such as “*protein X phosphorylates receptor Y*” (Torrecilla et al., 2007). However, these annotations often depend on identifying specific words such as the name of the gene, drug or protein, or specific phrases such as “opioid dependence” present in the text, or their variant forms or known synonyms from a dictionary, i.e., fairly simple design patterns (Castellini et al., 2012). In cognitive neuroscience, researchers seek to identify underlying neurobiological mechanisms, specifically relations between brain regions and mental functions. These include forward inferences, “*mental function X activates brain network Y*,” or reverse inferences, “*brain network Y is engaged during mental function X*” (Poldrack, 2011). The challenge for cognitive neuroscience is that the particular name of the mental function, experimental paradigm, or brain network often does not appear *per se* in the text, nor does any simple synonym because there is an inherent variance in how authors describe experimental design. Automated labeling of the concepts requires *inferring* the concepts from large and non-contiguous sections of the text. To that end, Neurosynth ^2^ (Yarkoni et al., 2011) was developed as an automated platform for archiving the results of neuroimaging articles, along with associated weightings of terms based on frequency of appearance in the articles’ abstracts. While this approach is capable of fast automated annotation of a substantial proportion of the literature, the annotations for a given article may lack sensitivity and specificity to relevant psychological constructs discussed in the article. An optimal platform would be one which utilizes the automated approach implemented in Neurosynth in conjunction with the structured vocabulary established by a more formalized ontology.

While initial progress has been made in developing an efficient and accurate machine learning classification approach for automated labeling on the abstracts of neuroimaging papers (Turner et al., 2013; Chakrabarti et al., 2014), a comprehensive assessment of predictive performance using different features and classifiers across abstracts or full article text has yet to be conducted. We therefore sought to expand our prior work by (1) developing a framework for automated annotation of neuroimaging publications, (2) evaluating classifier performance across a range of variable parameters (i.e., corpus, feature space, classification algorithm), and (3) characterizing relationships between labels by assessing the similarities between persistent vocabularies extracted from article text.

## Materials and Methods

### Corpora

In an effort to build an automated text-mining algorithm capable of classifying published neuroimaging articles, we utilized 2,633 articles from the BrainMap database ^3^ (Fox and Lancaster, 2002; Laird et al., 2005, 2009) that were published between 1992 and 2016 and their associated metadata labels derived by manual (i.e., human) annotation^3^. We extracted the text contained in the published abstracts using the PubMed API in Biopython ^4^. In addition, each neuroimaging publication was manually downloaded in PDF format, and the PDFMiner tool ^5^ was applied to extract full document text. Image-based PDFs were excluded from further analysis. This yielded the full text available in the manuscript, including title, authors, keywords, main body of the publication, and references, the totality of which includes text describing the study purpose, neuroimaging methodology, results, and interpretations of findings in using specific, author-determined terminologies. Thus, two text corpora were generated for this study (i.e., “abstracts-only” and “full-text”), which were separately analyzed to determine if similar knowledge can be extracted from succinct study descriptions as compared to the document as a whole.

### Metadata Labels

For automated article annotation, a classifier must be established using a training dataset with labeled articles. The Cognitive Paradigm Ontology (CogPO ^6^; Turner and Laird, 2012) is a taxonomy of labels utilized to represent experimental conditions based on the stimuli presented, the instructions given, and the responses requested. Each neuroimaging article was annotated with the established system of labels defined by CogPO. In total, there are 358 CogPO terms that are separated into distinct dimensions, including: *Behavioral Domain*, *Paradigm Class*, *Diagnosis, Context*, *Instruction*, *Stimulus Modality*, *Stimulus Type*, *Response Modality*, and *Response Type*. Typically, CogPO terms are assigned to experimental contrasts, which are defined by a reported set of activation (or deactivation) coordinates. *Behavioral Domain* describes the construct or mental process ostensibly isolated by the experimental contrast, according to the participant behaviors elicited during the performed task, the latter of which is described by a *Paradigm Class* term. *Diagnosis* refers to the participant population scanned during the neuroimaging study (including healthy individuals or participants with a disease or disorder), whereas *Context* describes what type of population effect was investigated (e.g., Disease Effects, Gender Effects, etc.). *Instruction* describes what the participant was instructed to do during the experiment, while *Stimulus Type* and *Modality* are descriptors for what stimuli were presented to the participants. Finally, *Response Modality* and *Response Type* describe the format for how the participant was instructed to overtly respond (if any), during the task. A complete list of all included CogPO terms is available in Supplementary Table 1.

### Manual Annotations

Each *experimental contrast* from the 2,633 neuroimaging publications archived in the BrainMap database was extracted, along with the set of metadata annotations derived from the CogPO labeling schema. Each experimental contrast was manually annotated by trained experts with a set of CogPO labels, and each publication may contain multiple experimental contrasts. Thus, in order to predict metadata label annotation for each publication, we collapsed all labels from each experimental contrast into one set of labels per neuroimaging article.

Importantly, the Behavioral Domain and Paradigm Class dimensions are organized hierarchically. For example, the Behavioral Domain *Cognition.Memory* includes two sub-types, *Cognition.Memory.Working* and *Cognition.Memory.Explicit*. Therefore, to enhance the ability of machine-learning classifiers to distinguish, at the highest level, between parent Behavioral Domains (i.e., *Action*, *Cognition*, *Emotion*, *Interoception*, *Perception*), we performed a hierarchical expansion procedure whereby all parent labels in a hierarchy, were assigned to the article in addition to the original label. For example, if a publication were assigned the Behavioral Domain *Cognition.Memory.Working*, it would have also been assigned the labels *Cognition.Memory* and *Cognition*. While Paradigm Classes do not necessarily have the same hierarchical structure across all labels, certain tasks do exhibit multiple variants, such as *Covert* and *Overt Word Generation*, and in such cases parent labels were assigned accordingly. To increase the power of certainty associated with label assignments using our machine-learning classifier, we only examined those labels with at least 80 instances (Figueroa et al., 2012) across neuroimaging publications. That is, if a specific metadata term, regardless of dimension, did not appear in at least 80 articles, it was not considered for assessment, reducing the total number of CogPO labels assessed from 358 to 86 (Supplementary Table 2).

We computed several descriptive measures pertaining to multi-label classification to provide reference for quantifying the variable range of label assignments to the neuroimaging articles. Label cardinality (*LC*_avg_) is the average number of labels per article. In addition to label cardinality, the minimum (*LC*_min_) and maximum (*LC*_max_) number of label assignments were calculated across all CogPO dimensions and for each dimension. Furthermore, label set proportions (Read et al., 2011) provide a reference for variability in label assignment across the articles and within dimensions. We subsequently calculated the proportion of unique label sets (*P*_uniq_) across all dimensions and for each dimension, as well as the proportion of the data that is assigned to the minimum (*P*_min_) and maximum (*P*_max_) number of labels.

### Analysis Pipeline

**FIGURE 1:**
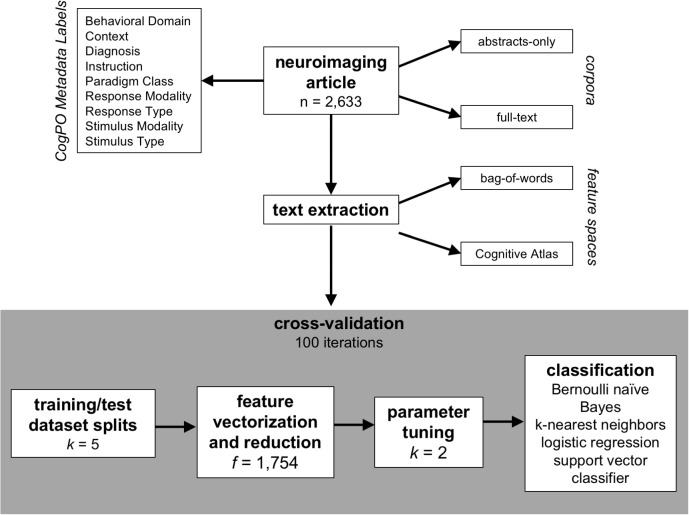
Analysis plan. The schematic describes the approach utilized in this analysis to identify the best classifier for automating the annotation process for published neuroimaging articles. The articles have been manually annotated to with metadata labels described by the Cognitive Paradigm Ontology (CogPO) and can be evaluated based on the text in the abstracts-only or the full extent of the article. The text can be extracted using the raw terms in the article (bag-of-words) or based on usage of terms from a defined vocabulary (Cognitive Atlas). Then, a repeated (100 iterations) cross-validation technique is performed for generating the classifiers where first the full dataset is split into five equally sized subsets, which are then split into training (80%) and testing (20%) datasets. The features (bag-of-words or Cognitive Atlas terms) are vectorized based on frequency of appearance and reduced to the only the most frequently used terms. Then, based on the specific classifier being used, the appropriate hyperparameters are tuned based on the training dataset, and then a classifier is constructed for each CogPO metadata label using the training dataset.

To evaluate classification accuracy and consistency across a combination of variable factors including corpora, features, and classifiers, we developed an analysis pipeline (Figure 1) combining tools available in the *Natural Language Toolkit* (NLTK ^7^; Loper and Bird, 2002; Bird et al., 2009) and machine learning algorithms from *scikit-learn* ^8^. For this purpose, we implemented a stratified, repeated cross-validation approach (Dietterich, 1998; Rodríguez et al., 2010) to ensure equal representation across folds, whereby for each combination of label, corpus, feature space, classification algorithm, and CogPO label, the binary classifier model was trained using an optimized set of parameters on the training dataset, and the subsequent predicted label was recorded for the test dataset. We evaluated classification accuracy by aggregating across macro F1-scores for each label across iterations. Then, we utilized a hierarchical clustering analysis to observe which Behavioral Domains and Paradigms Classes demonstrated similar representations of features selected for classification across iterations. For reference, all code utilized to perform these analyses are available on GitHub ^9^.

### Feature Space Definition

For each corpus investigated, we considered two *feature spaces*, for reducing the article text to terms (or features) used for classification purposes. In our analyses, the two types of features we used were defined by either “bag-of-words” or Cognitive Atlas terms, as described below.

#### Bag-of-Words

In the bag-of-words method, every whitespace character in the text indicated a separation of words, so every word with at least three letters can be considered a single feature through a process called *tokenization*. Given the complex description of psychological constructs and experimental design used in the neuroimaging literature, we also allowed for terms composed of one, two or three words (unigrams, bigrams, or trigrams). Any such combinations of terms were considered as potential features for the classification procedure. We also implemented an abbreviation expander ^10^, which was used to identify the corresponding terms associated with an abbreviation defined in the text. This procedure identified abbreviations appearing in parentheses and associated them with the terms appearing before the parentheses and whose letters began with the abbreviation letters. All instances of the abbreviation in the text were identified and replaced with the full term. This process served to provide consistency across article texts that are potentially representing similar information in different formats. Additionally, all non-alphanumeric characters (such as punctuation), except for hyphens, were removed from the text, and all terms using British-English spelling were converted to American-English spelling using a dictionary of spelling differences ^11^. An additional step for pre-processing the text included “stop word” removal. Commonly used terms that serve transitional or descriptive purposes, such as “the,” “and,” “are,” “at,” etc., are known as “stop words,” and are not beneficial for classification. We therefore filtered out the list of “stop words” provided by NLTK, available in the Supplementary Table 3. The final step for bag-of-words text pre-processing consisted of removing suffixes from terms such that each word was decomposed into its root form in a process called “stemming.” We again relied on the NLTK package and the English language *Snowball* stemmer (Bird, 2006) for this purpose. Here, the purpose of stemming was to establish consistency across terms that have the same meaning and root form but vary in the text based on usage. For example, the terms “viewing,” “viewed,” and “views” are all variants of the root “view,” but would be considered separate terms (and subsequently, features) if not for stemming procedure. During this transformation, the features for the classification procedure are now composed of lexical roots, which may or may not be a complete word.

#### Cognitive Atlas

The Cognitive Atlas ^12^ (Poldrack et al., 2011) is a collaboratively developed ontology for the field of cognitive science. The majority of items in the Cognitive Atlas are categorized as *Concepts*, *Tasks*, or *Disorders*, and have been developed by experts in the fields of psychology, cognitive science, and neuroscience. Furthermore, relationships between terms, called *assertions*, permit for a structured hierarchy that informs associations between psychological constructs and experimental manipulation. Although specialized relationships may exist within and between item categories, we limited feature weighting to Concept-Concept assertions; specifically, hypernym/hyponym (is-a). In a similar way that hierarchical expansion was performed for the metadata labels, we also implemented an ontological weighting schema between Cognitive Atlas terms defined by the “is-a” relationship (Poldrack, 2017; see text footnote 15 in “Software Dependencies” section). For example, if a Cognitive Atlas term appeared a given number of times in a document and is a “kind of” another Cognitive Atlas term, then the second term would be assigned the same count as the first term plus the count for the term itself. This weighting system was applied iteratively until the entirety of all term relationships was completed such that a term with multiple “is-a” relationships was influenced by the appropriate proportion of those term frequencies. In total, there are 1,744 terms in the Cognitive Atlas that describe *Concepts*, *Tasks*, or *Disorders*, along with 10 categories, for a total of 1,754 Cognitive Atlas features.

Text preprocessing for the “Cognitive Atlas” *feature space* was carried out in the same manner as the bag-of-words approach. The Cognitive Atlas provides not only a dictionary of relevant cognitive neuroscience terms, but also synonyms and alternate forms (e.g., “executive function” and “executive control”). Supplementing the Cognitive Atlas recommended alternate forms, we generated additional alternate forms of terms by removing hyphens and possessive apostrophes, moving parenthetical statements to the beginning of the term, and derived similar terms separated by a forward slash “/.” We additionally performed the “stemming” procedure as described above to reduce all Cognitive Atlas terms and their alternate forms to their roots.

### Feature Vectorization and Reduction

We transformed raw counts of feature (bag-of-words or Cognitive Atlas terms) appearance by calculating the term frequency-inverse document frequency (*tf-idf;* see Supplementary Material for a formal definition) for each feature in each article of the training-dataset. Specifically, the number of appearances of a given feature was extracted and sub-linearly scaled using *1 + log(tf)* to reduce the effect of high-frequency features, then multiplied by the inverse document-frequency to account for feature presence across articles. The inverse document-frequency values were smoothed by adding 1 to document frequencies to prevent zero divisions. Additionally, a threshold was imposed requiring a minimum frequency of 80 instances for each feature to reflect the minimum number of instances necessary for a metadata label to receive consideration for classification. That is, because we required a label to have a minimum of 80 instances, we also required a feature to appear at least 80 times. Then, only for the case of the “bag-of-words” *feature space*, if the total number of potential features for the classification procedure was greater than the number of Cognitive Atlas terms, a chi-square test was utilized to subsequently identify and eliminate the features that were irrelevant for classification. To this end, the chi-square tests measured dependence between all potential features, and the top 1,754 “bag-of-words” features that were *least likely* to be independent of class were retained. We chose to limit the number of bag-of-words terms to match the number of Cognitive Atlas terms to make the two feature spaces more directly comparable.

### Classifier, Parameter Tuning

We examined four different algorithms for classification, described below, to determine which approach produced the most reliable and accurate results. The performance of each classifier is dependent on the combination of different variables, or *hyperparameters*, that impact how the algorithm calculates the model for generating predictions. Each classifier is influenced by a unique set of *hyperparameters*. Thus, for each classifier, we performed a grid-search over different combinations of *hyperparameters* (from the classifier-specific set of *hyperparameters*) to determine which arrangement resulted in the most optimal classifier performance based on the training-dataset (Bergstra and Bengio, 2012). Then, once the optimal combination of *hyperparameters* was identified, the classifier and *hyperparameters* were used to generate predictions of metadata labels. This procedure was performed for each fold and each iteration, and the distributions of *hyperparameters* chosen for each classifier can be found in the Supplementary Table 4. Here, we briefly describe each classifier and the associated parameters chosen for tuning.

#### Bernoulli Naïve Bayes

The naïve Bayes algorithm is based on Bayes’ theorem with the assumption that each feature is independent. This classifier operates under the assumption that the probability of assigning a label to an article based on the specific *tf-idf* vector is proportional to the probability of that label occurring in the training-dataset multiplied by the union of probabilities of each feature’s association with that label (McCallum and Nigam, 1998; Metsis et al., 2006; Manning et al., 2008). Essentially, the probability that an article in the test-dataset is about a given label is calculated using the product of the probabilities of the features (that appeared in the test-dataset) in the training-dataset that were annotated with that label. Thus, this model is dependent on binary feature occurrence rather than frequency of occurrence. In the Bernoulli naïve Bayes approach, the non-occurrence of a feature is penalized, rather than ignored, in the calculation of the probability that a feature is associated with the label. If the resulting probability exceeds a threshold of 0.5, then it is assumed that the article in question is considered to be about the label being evaluated.

The only parameter that required tuning for the Bernoulli naïve Bayes classifier was the additive (Laplace/Lidstone) smoothing parameter, which primarily accounts for features which are not present in the training-dataset, preventing the occurrence of a zero probability for those features in further computations. The values for the smoothing parameter tested in the tuning grid-search were 0.01, 0.1, 1, and 10.

#### Support Vector Classifier

Support vector machines construct a hyperplane in high-dimensional space that separates data-points according to binary classification (is or is not annotated with the label), where the optimal separation is achieved when the hyper-plane is maximally distant from the nearest training data-points of different classes (the maximum-margin hyperplane). In classification, the hyper-plane is constructed to separate articles in the *tf-idf* matrix that were or were not about a given label, after transformation by a radial basis function kernel which allows the feature space to be non-linear (Smola and Schölkopf, 2004). Put another way, the radial basis kernel function (defined in the Supplementary Text) incorporates a Gaussian function to calculate the distance between feature vectors.

The parameters that required tuning for the support vector classifier were the penalty of the error term and kernel coefficient for the kernel function. For the *radial basis function* kernel, the error term trades misclassification of training examples against the simplicity of the decision surface, and the kernel coefficient defines the extent to which a single article in the training-dataset influences the classifier. The error terms used for tuning in the grid-search were 1, 10, and 100, and the potential kernel coefficients were 0.01, 0.1, and 1.

#### Logistic Regression

The logistic regression algorithm is a classification algorithm based on generalized linear models, where the probabilities that a given article is about a label is modeled using a logistic function (Yu et al., 2011). In the current approach, a binary classifier is independently developed for every label where the model coefficients corresponding to each feature in the training-dataset are calculated to minimize the error using a cost function. The LIBLINEAR library utilizes a coordinate descent algorithm to optimize the regression model (Fan et al., 2008). *tf-idf* weights from the testing-dataset article are entered into the resulting regression model, and the log-odds is then modeled as a probability using the logistic function.

The parameters tuned in the grid-search accounted for the regularization strength and the function for penalty normalization. Regularization in machine learning is a term that prevents the model from overfitting to the training-dataset, and the lower the regularization, the more likely overfitting is to occur. Penalty normalization essentially adds either square loss or absolute deviation loss of the magnitude of the coefficients to the penalty term of the cost function. The regularization strengths submitted for tuning were 0.01, 0.1, 1, 10, and 100; and the penalty normalization functions were the *L1-norm* or the *L2-norm*.

#### K-Nearest Neighbors

The kNN algorithm identifies the *k* articles in the training-dataset closest in distance between their respective *tf-idf* vectors and that of the test-article to be classified. That is, the distance between all *tf-idf* vectors in the training-dataset and the article to be classified was calculated using the appropriate distance metric, and the *k* articles with the smallest distance were identified. Then, a majority vote is calculated from those *k-nearest* articles to determine if the test-article should be annotated with a given label. In this instance, if more of the *k-nearest* articles are not classified with the label under consideration, then the model will not predict that label for the given article.

The kNN algorithm is dependent on the chosen *k*, the distance metric, and distance weighting for predictions. Our parameter-tuning grid-search operated on *k* = 1, 3, 5, 7, 9; calculated distances between *tf-idf* vectors in the training- and test-dataset, which have equivalent lengths (i.e., number of features) using both the Manhattan and Euclidean distance algorithms; and based predictions on uniform and weighted distances. Uniform distances indicated that all points in a neighborhood were weighted equally, whereas points could also be weighted by the inverse of their distance. In this case, closer neighbors of a query point had a greater influence than neighbors that were further way. As the input datasets are large and the kNN classification approach requires all the data available, distance calculation algorithms can be used to identify the nearest neighbors. The algorithm (BallTree, KDTree, brute-force) used to compute the nearest neighbors were automatically determined based on the sparsity of the inputs (Bently, 1975; Omohundro, 1989).

### Classifier Training

For the unique combination of a given metadata label, corpora (“abstract-only” or “full-text”), and feature space (“bag-of-words” or “Cognitive Atlas”), a repeated five-fold cross-validation procedure was performed 100 times. In this scheme, for each iteration, the publications were first randomly split into five groups. Then, within the iteration, each of the groups was selected as the test dataset once (and the other four were combined into a training dataset). The *tf-idf* vectorization and feature reduction techniques described above were subsequently performed for the training-datasets in each fold and each iteration to increase generalizability of the model and improve learning performance (Tang et al., 2013). For the bag-of-words feature space, the vocabulary (i.e., the set of unigrams, bigrams, and trigrams extracted from the text and used to train the classifier) was defined independently based on the fold’s training dataset, while for the Cognitive Atlas feature space the vocabulary was already defined. Bag-of-words features derived from the training dataset or Cognitive Atlas terms were then subjected to a similar *tf-idf* vectorization procedure in the test-dataset. This resulted in two independent matrices with dimensions equal to the number of features derived from the training-dataset and number of articles in the training-dataset and test-dataset, respectively (Manning et al., 2008; Baeza-Yates and Ribeiro-Neto, 2011). The procedure outlined above, consisting of vectorization, feature reduction, and classifier training/testing was performed five times for each of the 100 iterations which were performed for each combination of *feature space*, *corpus*, and *classifier* for a total of 8,000 permutations for each CogPO label. Within each iteration and fold, classifiers were then trained using the training-dataset *tf-idf* feature matrix, and predictions for articles in the test-dataset were made using the test-dataset *tf-idf* feature matrix as input.

### Evaluation

#### F1-Scores

To build and assess classifier performance in assigning CogPO labels to neuroimaging articles, we explored two *corpora* (“abstracts-only” and “full-text”), two *feature spaces* (“bag-of-words” and “Cognitive Atlas”), and four *classifiers* (“Bernoulli naïve Bayes,” “support vector classifier,” “logistic regression,” and “k-nearest neighbors”). Classifiers for each label were modeled using a repeated cross-validation procedure, whereby for each of the 100 iterations, the neuroimaging articles and associated labels were split into five training- and test-datasets (thus producing 500 estimates of classifier performance per label and per combination of corpus, feature space, and classifier). Macro F1-scores (see Supplementary Text for F1-score derivation) were used as the standard measure of classifier performance and calculated for each iteration for each label so that our results were not biased toward the most frequently occurring metadata labels within and across dimensions (Sokolova and Lapalme, 2009). For Macro-F1 calculation, F1 = 2 × $\frac{\text{precision}\times \text{recall}}{\text{precision}+\text{recall}}$ precision = $\frac{\text{tp}}{\text{tp}+\text{fp}}$ recall = $\frac{\text{tp}}{\text{tp}+\text{fn}}$ the mean and standard deviation of F1-scores across iterations and folds provided average levels of performance and consistency of performance for each label. Then, to assess classifier performance for each CogPO dimension, the mean and standard deviation of F1-scores were calculated across iterations and folds for all labels within a dimension. Additionally, we calculated Micro F1-scores to obtain a characterization of classifier performance that does not over-emphasize classes that are under-represented while under-emphasizing classes that are over-represented. For Micro F1-score calculation, F1-scores were calculated across all labels within a CogPO dimension for each combination of corpora, feature space, and classifier, and averaged across iterations. Both Macro and Micro F1-scores can range from 0, the worst score possible, and 1, for perfect precision and recall.

#### Baseline Performance Estimation

To compare the classifiers, we calculated the level of performance one would expect based on simply choosing the most frequently occurring metadata labels, derived using each combination of parameters. To do this, Macro F1-scores were calculated for a pseudo-prediction matrix that was artificially generated by “predicting” that all articles were annotated with the metadata labels within each dimension that occurred most frequently across the dataset. First, the average label cardinality (*LC*_avg_) for each dimension was used to select the (rounded) *LC*_avg_ most frequently occurring metadata labels. Then, the pseudo-prediction matrix was filled in with a value of 1 for all articles using those selected metadata labels for each dimension. F1-scores were calculated using this “prediction matrix” to obtain a baseline level of classifier performance.

#### Hierarchical Recall and Precision

Additional metrics for evaluating classifier performance are hierarchical recall and precision. Due to the hierarchical nature of Behavioral Domains in CogPO and the current implementation of hierarchical expansion for label assignment, we explored evaluating these metrics to assess classifier performance. The purpose for evaluating hierarchical recall and precision is to determine the performance of predicting the parent label (e.g., Cognition.Memory) when an article is also predicted to have been annotated with one of its child domains (e.g., Cognition.Memory.Working). However, the current classification problem is one that generates binary classification models, and therefore label predictions are independent of one another. That is, classifiers for Cognition.Memory and Cognition.Memory.Working are trained, predicted, and evaluated independently of one another across five-fold and 100 iterations for each combination of corpora, feature space, and classifier. Nonetheless, we derived hierarchical recall and precision metrics for hierarchical Behavioral Domain labels *within* iterations, and averaged over all iterations and Behavioral Domain labels.

### Feature Similarity Across Labels

The *bag-of-words* approach uses the most frequently appearing one-, two-, or three-word terms across all articles annotated with a given label for features when generating a classification algorithm. Within each fold across iterations of the classification procedure, we chose to use the top 1,754 features for each label from the *bag-of-words*, the same number of Cognitive Atlas features, so that each feature space would be comparable in size. We sought to determine if, across folds and iterations, different sets of features from the *bag-of-words* approach were more frequently used for classification across the CogPO dimensions *Behavioral Domain* and *Paradigm Class*. First, we calculated the average feature frequency for a given label within the “full-text” corpora and “logistic regression” classifier combination as it performed the best across the possible permutations when using Macro F1-scores as a proxy for classification performance. Then, we calculated the Spearman correlation coefficient between each possible pairing of feature frequency distributions from *Behavioral Domain* and *Paradigm Class* labels. To control for correlations that are influenced by labels that tend to be annotated together, we regressed the frequency of co-occurrence [as estimated by the Dice Similarity Index (Dice, 1945)], such that the resulting residuals represented a true similarity between the labels’ feature distributions. Hierarchical clustering was then applied to the resulting cross-correlation matrix (Laird et al., 2015; Riedel et al., 2018) using the “correlation distance” and “weighted linkage” metrics in the MATLAB (Natick, MA, United States) computing environment to observe how similar labels were classified based on similar sets of terms.

The resulting clusters of labels from the hierarchical clustering analysis serve as a proxy for demonstrating how articles assigned with similar labels tend to use similar vocabulary. To demonstrate this effect, we then sought to present the most consistently utilized features across iterations for each cluster. As indicated above, before the classifiers were determined, the feature set for each label and each iteration was reduced from the full bag-of-words to the top 1,754 features. We calculated the mean occurrence of each feature across labels within a cluster and utilized the top ten percent of those bag-of-words features and their corresponding frequencies to generate a “word cloud” visualization^13^. In this representation, the features exhibiting the highest frequency across labels in a cluster appear in larger font sizes in the word cloud.

## Results

A collection of 2,633 neuroimaging articles and their associated labels derived from the CogPO vocabulary were submitted to a repeated cross-validation technique to determine which combination of corpora, features, and classifier resulted in an optimal performance of automated article labeling. A total of 100 iterations of five-fold cross-validation were performed for each combination and label. Average predictive performance was assessed using the mean of Macro F1-scores across iterations and folds, and performance consistency was assessed using the standard deviation of Macro F1-scores for across iterations and folds. As indicated above, we utilized Macro F1-scores as our measure of performance such that our results would not be biased toward the most frequently occurring labels.

### Labels

Our classification analysis included 26 Behavioral Domains, 17 Paradigm Classes, 3 Context terms, 5 Diagnoses, 12 Instructions, 4 Stimulus Modalities, 12 Stimulus Types, 3 Response Modalities, and 4 Response Types. Multi-label classification metrics, such as label cardinality and label set proportions, provide a means for interpreting the variable range of true label annotations to the neuroimaging articles. The average, minimum, and mean label cardinality and set proportions were calculated across all CogPO dimensions and for each dimension (Table 1). On average, each neuroimaging article was annotated with ∼12 labels across all CogPO dimensions, while one article was annotated with only one label (the minimum), and two articles were annotated with 37 labels (the maximum). Although there are nine dimensions in CogPO, the reason that one neuroimaging article was only annotated with one label is because the other annotated labels did not occur in at least 80 instances across the entire neuroimaging corpora. The number of unique combinations of label assignments across CogPO dimensions was about 87% of the total dataset, indicating a diversity of experimental designs across the neuroimaging corpora. When considering the individual CogPO dimensions, on average, each neuroimaging article was assigned approximately three Behavioral Domains, whereas all other dimensions were assigned on average about 1–1.5 labels. As previously mentioned, the minimum number of label assignments across all dimensions was 0. This occurred the most frequently in the Paradigm Class dimensions, where roughly 29% of the neuroimaging articles were not assigned a label. It is also worth noting that every neuroimaging article had *at least* one label assignment after thresholding.

**Table 1:** Label cardinality and set proportions.

Dimension	Behavioral domain	Context	Diagnosis	Instruction	Paradigm class	Response modality	Response type	Stimulus modality	Stimulus type	Overall
*LC*_avg_	2.96	0.98	1.02	1.52	0.85	1.27	1.22	1.19	1.40	12.41
*LC*_min_	0	0	0	0	0	0	0	0	0	1
*LC*_max_	13	3	3	6	4	3	3	4	7	37
*P*_uniq_	12.19	15.34	0.61	7.41	5.36	0.30	0.57	0.61	6.84	86.86
*P*_min_	2.16	16.82	6.68	2.96	29.05	1.14	2.73	1.48	6.27	0.04
*P*_max_	0.04	0.61	0.49	0.15	0.19	0.76	0.76	0.08	0.04	0.08

*Label cardinality metrics, such as the average (LC_avg_), minimum (LC_min_), and maximum (LC_max_) number of labels assigned to a neuroimaging article, were calculated for each Cognitive Paradigm Ontology dimension and across all dimensions. These metrics were derived using the known manual annotations. Additionally, label set proportions were calculated, such as the proportion of articles assigned with the minimum (P_min_) or maximum (P_max_) number of labels and the proportion of unique label set combinations (P_uniq_) across all neuroimaging articles.*

### Evaluation

#### Overall Performance

**FIGURE 2:**
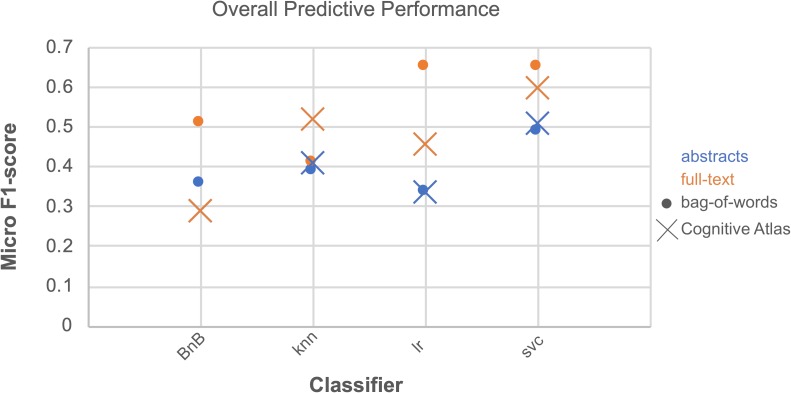
Overall predictive performance across classifiers. Predictive performance evaluated as the average of Micro F1-scores for each combination of parameters over all CogPO dimensions provides an outlook of comparative performances. The combination of parameters with the highest performance occurred for *bag of words*, *full-text*, and *logistic regression*; however, *bag of words*, *full-text*, and *support vector classifier* performed nearly equivalently. Performance levels for the Bernoulli naïve Bayes classifier, Cognitive Atlas feature space and full-text and abstract-only analyses were the same, indicating why it does not appear in the current figure.

We ran an overall ANOVA to test for differences in Macro F1-scores when considering different parameters and combinations of parameters for classification (Figure 2 and Table 2). Two findings emerge from this analysis: that the interaction between the three parameters we tested indicated results will significantly vary depending on the *corpus*, *feature space*, and *classifier* chosen for article annotation, and importantly, that performance does not vary across those parameters when considering CogPO dimensions. This second point suggests that different classification parameters are NOT needed when annotating Behavioral Domains and Paradigm Classes, for instance.

**Table 2:** ANOVA results.

ANOVA results	*F*	*df*	Pr(>*F*)	Significance
Dimension	3.734	8	0.000976	^∗∗∗^
Classifier	128.301	3	<2E-16	^∗∗∗^
Dimension × classifier	1.758	24	0.0187	^∗^
Corpora	104.061	1	5.99E-16	^∗∗∗^
Dimension × corpora	0.242	8	0.981	
Feature	20.504	1	2.14E-05	^∗∗∗^
Dimension × feature	2.065	8	0.0496	^∗^
Classifier × corpora	52.56	3	<2E-16	^∗∗∗^
Dimension × classifier × corpora	0.68	24	0.869	
Classifier × feature	85.522	3	<2E-16	^∗∗∗^
Dimension × classifier × feature	2.142	24	0.00214	^∗∗^
Corpora × feature	34.221	1	1.13E-07	^∗∗∗^
Dimension × corpora × feature	1.124	8	0.357	
Classifier × corpora × feature	45.15	3	<2E-16	^∗∗∗^
Dimension × classifier × corpora × feature	0.98	24	0.493	

*An overall ANOVA test was performed to test differences between F-scores for each potential combination of parameters tested in our analysis. Interactions between parameters were also included to inform the effect of different combinations parameters on the resulting F-scores. ^∗∗∗^p < 0.001, ^∗∗^p < 0.01, ^∗^p < 0.05.*

With respect to CogPO dimensions, *Diagnosis* labels demonstrated the highest performance and *Stimulus Type* labels demonstrated the most consistent performance across iterations (Supplementary Table 5). To provide insight into classification performance at different levels of combinations of the parameters varied, first we examined which combinations of *corpora, feature space*, and *classifier* independently performed the best (Supplementary Table 6). On average, when only considering *corpus*, “full-text” out-performed “abstracts” and was the most consistent. When only considering *feature space*, the “bag-of-words” approach out-performed the “Cognitive Atlas” and was the most consistent; and when only considering the *classifiers*, “support vector classifiers” out-performed all others and was most consistent. Second, we examined which combination of parameters yielded the highest performance. We observed that the combination of “full-text” and “support vector classifiers” out-performed all other combinations of *corpus* and *classifier*, and was the most consistent; the combinations of “bag-of-words” and “support vector classifiers” out-performed all other combinations of *feature space* and *classifier*, and was the third-most consistent; and “full-text” and “bag-of-words” out-performed all other combinations of *corpus* and *feature space*, and was the most consistent. Interestingly, when considering “abstracts-only,” the “bag-of-words” and “Cognitive Atlas” *corpora* performed almost equivalently, with “bag-of-words” performing slightly better. Third, we examined which combination performed the best across all three parameters. We observed that the average levels of performance were highest according to Macro F1-scores across all CogPO dimensions (Table 3) for the combination of “full-text,” “bag-of-words,” and the “logistic regression” classifier, though the performance for “full-text,” “bag-of-words,” and “support vector classifier” was not substantially different enough to indicate one approach is truly superior to the other. However, the subsequent ancillary analyses focus on the “logistic regression” classifier since it did perform the best. With respect to Micro F1-scores (Table 4), the combination of “full-text,” “bag-of-words,” and the “support vector classifier” performed best, though not substantially better than the same combination when using the “logistic regression.” Thus, across evaluation metrics (Macro/Micro F1-scores) performance was always highest when using the “full-text” corpus, “bag-of-words” feature space, and either the “logistic regression” classifier or “support vector classifier.”

**Table 3:** Macro F1-scores for combinations of variables.

Dimension	Behavioral domain	Context	Diagnosis	Instruction	Paradigm class	Response modality	Response type	Stimulus modality	Stimulus type	Overall
**Baseline**										
	0.06	0.31	0.21	0.09	0.01	0.49	0.36	0.30	0.07	
**Corpora + feature space + classifier**										
**Abstract-only + bag-of-words**										
Bernoulli naïve Bayes	0.38 (0.26)	0.62 (0.32)	0.45 (0.26)	0.28 (0.23)	0.26 (0.21)	0.57 (0.20)	0.45 (0.27)	0.53 (0.20)	0.27 (0.19)	0.36 (0.26)
k-nearest neighbor	0.45 (0.25)	0.61 (0.36)	0.65 (0.32)	0.24 (0.22)	0.33 (0.24)	0.54 (0.27)	0.42 (0.30)	0.57 (0.24)	0.21 (0.21)	0.39 (0.28)
Logistic regression	0.43 (0.33)	0.58 (0.41)	0.69 (0.34)	0.12 (0.25)	0.28 (0.32)	0.49 (0.33)	0.36 (0.37)	0.55 (0.34)	0.15 (0.27)	0.34 (0.36)
Support vector classifier	0.56 (0.23)	**0.69 (0.31)**	**0.73 (0.30)**	**0.33 (0.24)**	**0.44 (0.23)**	**0.61 (0.19)**	**0.50 (0.25)**	**0.66 (0.19)**	**0.31 (0.21)**	**0.49 (0.27)**
**Abstract-only + Cognitive Atlas**										
Bernoulli naïve Bayes	0.38 (0.31)	0.52 (0.38)	0.45 (0.31)	0.16 (0.22)	0.22 (0.27)	0.46 (0.33)	0.35 (0.35)	0.49 (0.31)	0.09 (0.19)	0.29 (0.31)
k-nearest neighbor	0.51 (0.20)	0.51 (0.37)	0.57 (0.29)	0.30 (0.18)	0.38 (0.24)	0.52 (0.26)	0.43 (0.27)	0.55 (0.22)	0.18 (0.15)	0.41 (0.25)
Logistic regression	0.44 (0.30)	0.53 (0.38)	0.68 (0.34)	0.20 (0.27)	0.29 (0.35)	0.48 (0.34)	0.42 (0.32)	0.51 (0.32)	0.06 (0.17)	0.34 (0.34)
Support vector classifier	**0.57 (0.18)**	**0.62 (0.28)**	**0.72 (0.27)**	**0.41 (0.15)**	**0.50 (0.21)**	**0.60 (0.20)**	**0.52 (0.23)**	**0.62 (0.18)**	**0.32 (0.15)**	**0.51 (0.22)**
**Full-text + bag-of-words**										
Bernoulli naïve Bayes	0.54 (0.17)	0.70 (0.28)	0.61 (0.15)	0.41 (0.16)	0.46 (0.15)	0.64 (0.17)	0.57 (0.21)	0.64 (0.16)	0.39 (0.13)	0.51 (0.18)
k-nearest neighbor	0.46 (0.21)	0.58 (0.27)	0.69 (0.20)	0.28 (0.19)	0.36 (0.21)	0.64 (0.19)	0.49 (0.30)	0.59 (0.16)	0.25 (0.15)	0.41 (0.24)
Logistic regression	**0.70 (0.13)**	0.85 (0.10)	0.87 (0.08)	**0.51 (0.19)**	**0.63 (0.19)**	0.76 (0.09)	0.63 (0.26)	**0.77 (0.13)**	**0.51 (0.23)**	**0.65 (0.20)**
Support vector classifier	0.69 (0.14)	**0.86 (0.10)**	**0.87 (0.09)**	0.51 (0.17)	0.62 (0.19)	**0.77 (0.09)**	**0.66 (0.21)**	0.77 (0.14)	0.50 (0.21)	0.65 (0.20)
**Full-text + Cognitive Atlas**										
Bernoulli naïve Bayes	0.37 (0.28)	0.55 (0.39)	0.64 (0.19)	0.12 (0.22)	0.22 (0.24)	0.49 (0.33)	0.40 (0.33)	0.41 (0.35)	0.07 (0.17)	0.29 (0.31)
k-nearest neighbor	0.61 (0.18)	0.64 (0.29)	0.71 (0.18)	0.39 (0.18)	0.50 (0.19)	0.64 (0.17)	0.52 (0.25)	0.64 (0.20)	0.29 (0.21)	0.52 (0.23)
Logistic regression	0.58 (0.28)	0.58 (0.41)	0.85 (0.07)	0.21 (0.27)	0.46 (0.31)	0.61 (0.24)	0.49 (0.32)	0.58 (0.35)	0.17 (0.26)	0.46 (0.34)
Support vector classifier	**0.67 (0.14)**	**0.73 (0.22)**	**0.87 (0.07)**	**0.49 (0.15)**	**0.60 (0.19)**	**0.69 (0.13)**	**0.60 (0.21)**	**0.71 (0.16)**	**0.39 (0.19)**	**0.60 (0.21)**

*Performance measures for each possible combination of options from all potential variables, as indicated by average and standard deviations of the Macro F1-scores across iterations for each Cognitive Paradigm Ontology dimension. Bolded values indicate the classifier that demonstrated the best performance across iterations for labels within the CogPO dimension for the respective combination of parameters.*

**Table 4:** Micro F1-scores for combinations of variables.

Dimension	Behavioral domain	Context	Diagnosis	Instruction	Paradigm class	Response modality	Response type	Stimulus modality	Stimulus type	Overall
**Baseline**										
	0.06	0.31	0.21	0.09	0.01	0.49	0.36	0.30	0.07	
**Corpora + feature space + classifier**										
**Abstract-only + bag-of-words**										
Bernoulli naïve Bayes	0.57 (0)	0.64 (0)	0.83 (0)	0.85 (0)	0.8 (0)	0.86 (0)	0.47 (0)	0.38 (0)	0.36 (0)	0.61 (0.16)
k-nearest neighbor	0.61 (0)	0.63 (0)	0.84 (0)	0.81 (0.01)	0.85 (0.01)	0.85 (0)	0.38 (0)	0.4 (0)	0.33 (0.01)	0.64 (0.19)
Logistic regression	0.63 (0)	0.68 (0)	0.88 (0)	0.89 (0.01)	0.89 (0.01)	0.91 (0)	0.43 (0)	0.5 (0)	0.4 (0.01)	0.63 (0.19)
Support vector classifier	0.62 (0.01)	0.68 (0.01)	0.84 (0)	0.83 (0)	0.89 (0.01)	0.89 (0)	0.42 (0)	0.49 (0)	0.44 (0.01)	0.68 (0.17)
**Abstract-only + Cognitive Atlas**										
Bernoulli naïve Bayes	0.62 (0)	0.75 (0)	0.83 (0)	0.91 (0.01)	0.8 (0.01)	0.91 (0)	0.52 (0)	0.61 (0)	0.46 (0)	0.58 (0.21)
k-nearest neighbor	0.59 (0)	0.72 (0)	0.85 (0)	0.86 (0.01)	0.87 (0.01)	0.89 (0)	0.36 (0)	0.45 (0.01)	0.34 (0.01)	0.62 (0.19)
Logistic regression	0.6 (0)	0.77 (0)	0.84 (0)	0.92 (0)	0.9 (0)	0.93 (0)	0.46 (0)	0.61 (0)	0.41 (0.01)	0.60 (0.20)
Support vector classifier	0.71 (0)	0.75 (0.01)	0.87 (0)	0.89 (0.01)	0.9 (0.01)	0.92 (0)	0.5 (0)	0.57 (0.01)	0.54 (0)	0.66 (0.15)
**Full-text + bag-of-words**										
Bernoulli naïve Bayes	0.44 (0)	0.68 (0)	0.69 (0)	0.66 (0)	0.69 (0)	0.71 (0)	0.76 (0)	0.39 (0)	0.33 (0)	0.65 (0.14)
k-nearest neighbor	0.41 (0.01)	0.67 (0.01)	0.7 (0)	0.65 (0.01)	0.68 (0.01)	0.7 (0)	0.73 (0.01)	0.23 (0.01)	0.21 (0.02)	0.64 (0.20)
Logistic regression	0.51 (0)	0.71 (0)	0.72 (0)	0.69 (0)	0.71 (0)	0.77 (0)	0.8 (0)	0.34 (0)	0.39 (0)	0.76 (0.11)
Support vector classifier	0.53 (0)	0.68 (0)	0.71 (0)	0.67 (0)	0.7 (0)	0.73 (0)	0.77 (0)	0.28 (0)	0.4 (0)	0.77 (0.12)
**Full-text + Cognitive Atlas**										
Bernoulli naïve Bayes	0.66 (0)	0.72 (0)	0.8 (0)	0.71 (0)	0.79 (0.01)	0.75 (0)	0.85 (0)	0.45 (0)	0.59 (0.01)	0.59 (0.22)
k-nearest neighbor	0.56 (0)	0.69 (0)	0.74 (0)	0.68 (0)	0.73 (0.01)	0.71 (0)	0.79 (0)	0.22 (0)	0.37 (0)	0.69 (0.16)
Logistic regression	0.65 (0)	0.75 (0)	0.81 (0)	0.73 (0.01)	0.8 (0.01)	0.79 (0)	0.86 (0)	0.31 (0)	0.58 (0)	0.68 (0.17)
Support vector classifier	0.63 (0)	0.74 (0)	0.77 (0)	0.72 (0.01)	0.75 (0)	0.8 (0)	0.83 (0)	0.42 (0)	0.5 (0)	0.74 (0.13)

*Performance measures for each possible combination of options from all potential variables, as indicated by average and standard deviations of the Micro F1-scores across iterations for each Cognitive Paradigm Ontology dimension.*

#### Baseline Performance Estimation

Our baseline performance estimation in which Macro F1-scores were calculated for a pseudo-prediction matrix yielded values for comparing our classifiers performance. In a few instances, some combinations of *corpus*, *feature space*, and *classifier* failed to outperform the baseline performance estimation for the CogPO dimensions *Response Modality* and *Response Type*. However, the best performing combination of parameters for each dimension *always* outperformed the baseline performance estimations.

#### Hierarchical Recall and Precision

Generally speaking, across all combinations of corpora, feature space, classifier, and Behavioral Domain labels, hierarchical recall was roughly 0.55, while hierarchical precision was 0.71. This difference between recall and precision indicates that more false negatives were identified than false positives, meaning articles annotated with a sub-label were not as frequently classified with the associated parent-label. This is not unexpected as feature differentiation among the parent label is greater and non-specific compared to the sub-label. Hierarchical recall and precision distributions calculated for each Behavioral Domain assessed across every combination of corpora, feature space, and classifier can be found in Supplementary Figures 2, 3, respectively.

**FIGURE 3:**
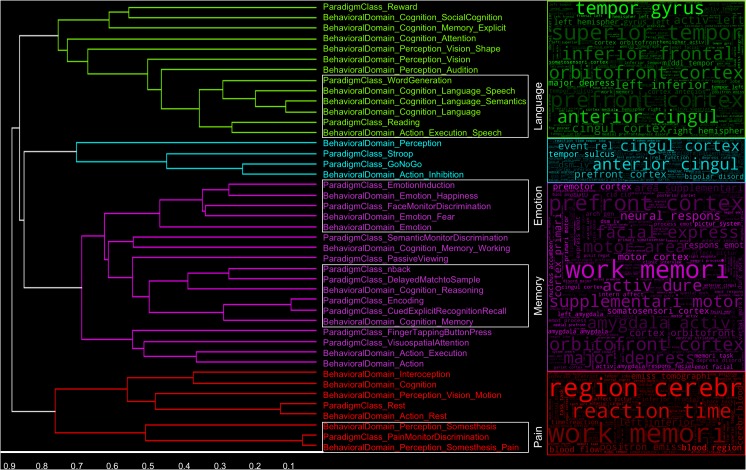
Label similarity dendrogram. Similarity between Behavioral Domain and Paradigm Class metadata labels based on features selected for classification across folds and iterations. Clusters are representative of labels and their corresponding manuscript in which similar language was used throughout the whole text. The associated “word clouds” were generated by using the top 10% of the most frequently used bag-of-words features across labels and iterations in each cluster.

### Feature Similarity Across Labels

We implemented a hierarchical clustering analysis on a matrix of residual correlation coefficients between pairwise Behavioral Domain and Paradigm Class label feature representation distributions to observe which labels tended to demonstrate higher similarities of terms usage in the “full-text” (Figure 3) and “abstracts-only” (Supplementary Figure 4) extracted from neuroimaging articles. We chose an arbitrary clustering threshold based on visual inspection of the resulting dendrogram to relate CogPO labels assigned to individual clusters. We observed four clusters of CogPO labels in the dendrogram and their corresponding word clouds indicate not only which features were most consistently used across classifiers for each label in a cluster, but also represent an associated vocabulary respective to the constructs in each cluster. A persistent observation across all word clouds is the inclusion of a number of brain anatomy, structure, or location descriptors such as “anterior cingul” (anterior cingulate), “cingul cortex” (cingulate cortex), and “left amygdala.” Furthermore, terms corresponding to mental constructs such as “work memori” (working memory), “intern affect” (internal affect), and “express emot” (express emotion), coupled with experimental design descriptions like “event rel” (event related) and “pictur system” (picture system) provide a broad overview of psychological systems interrogated across a large set of studies. Additionally, diagnoses such as “major depress” (major depressive disorder) and “bipolar disord” (bipolar disorder) can provide insight into either the neural systems most *studied* in specific patient populations or the neural systems most *affected* in specific patient populations. Finally, journal titles and author names are also represented in these word clouds indicating specific emphases on certain topics by journals (which may be subsequently biased due to study inclusion in this analysis) or domain of study for different principal investigator’s labs.

**FIGURE 4:**
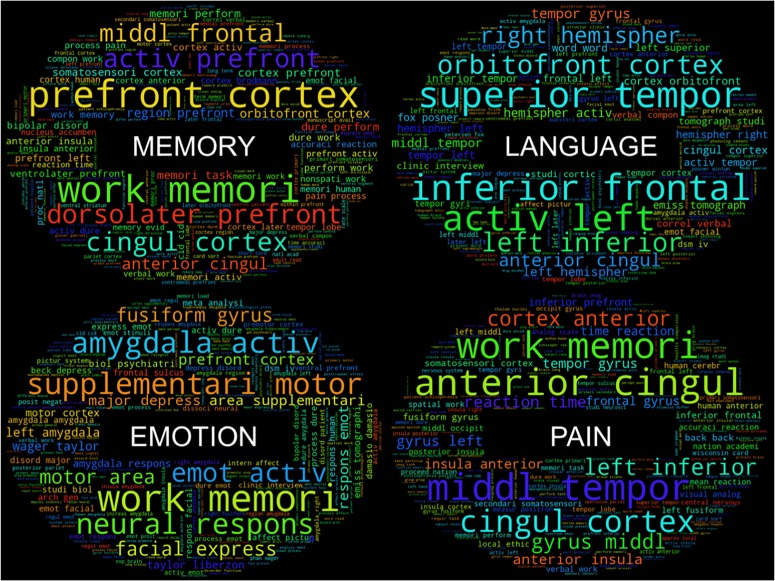
Feature “word clouds” from cluster subsets. “Word clouds” for the four subsets of clusters were generated by utilizing the top 10% of the most frequently used bag-of-words features across labels in each subset. Larger words indicate a larger representation of feature frequency within each distribution.

As a purely exploratory investigation, within these primary clusters, individual groupings of labels that are combinations of Behavioral Domain and Paradigm Classes emerge that represent similar psychological constructs. For instance, in one cluster (red), a grouping of the Behavioral Domain labels “Perception.Somesthesis” and “Perception.Somesthesis.Pain” and Paradigm Class label “Pain Monitor/Discrimination” represent a very specific subset of functional neuroimaging studies investigating the neural responses to “pain.” Further high-level psychological constructs that can be identified by the dense grouping of similar CogPO labels include “Memory,” “Emotion,” and “Language.” Following the same procedure for generating word clouds corresponding to each cluster, we additionally created word clouds for each psychological construct to determine if specific terminology in each sub-grouping would yield a more informative knowledge base for describing these paradigms. The word clouds (Figure 4) associated with these individual sub-groupings of labels provide an even more fine-grained assessment of the most frequently used features in these inferred topics with terms such as “nonspati work” (non-spatial working), “verbal work” (verbal working), “term memori” (term memorization) in the “Memory” subset and “facial express” (facial expression), “fusiform gyrus,” and “amygdala activ” (amygdala activation) in the “Emotion” subset.

## Discussion

Neuroimaging meta-analyses for knowledge modeling are becoming increasingly prevalent due to the increasing rate and number of publications. Curating and synthesizing this data is time consuming, subjective, and prone to errors of omission simply because the scientific literature is too large. We utilized 2,633 neuroimaging articles to determine the most optimal combination of corpus (*abstract*, *full-text*), feature (*bag-of-words*, *Cognitive Atlas*), and classifier (*Bernoulli naïve Bayes, support vector classifier, logistic regression, k-nearest neighbors*), that resulted in the highest predictive performance. Our findings indicate that if CogPO labels are to be used for synthesizing neuroimaging articles and full-article text is available, using the *bag-of-words* feature space and the *logistic regression* classifier will provide optimal performance of article annotation, though it only slightly outperformed the full-text, bag-of-words, and support vector classifier combination, whereas if only article abstracts are available, the *Cognitive Atlas* feature space and *support vector* classifier should be used. These recommendations are expanded upon in the ensuing discussion.

### Full-Text vs. Abstracts

We sought to evaluate whether classifiers performed better when using the text from the entire neuroimaging article or just the article abstract. The motivation for performing this assessment was based on the idea that short, concise descriptors in article abstracts would be used to convey psychological constructs and experimental design, whereas phrases and terminology describing the study design would be captured by using full article text. Previous research has illustrated techniques utilized for document classification and short-text classification (e.g., Turner et al., 2013) and we identified one paper (Bui et al., 2016) which attempted to classify text patterns according to which section of an article it appeared in (i.e., title, abstract, text-body, etc.). In addition, within the context of text-mining in genetics literature, structural differences existed between abstract-only and full article text, with longer sentences and increased parenthesized material in the article text (Cohen et al., 2010). Cohen et al. (2010) additionally found that semantic classes (corresponding to gene, mutation, disease, and drug) exhibited differential densities in article and abstract text, yielding the potential for characterizing articles based on densities of CogPO dimensions across sections of the article. Overall, across all feature spaces and classifiers, predictive performance was higher when using text extracted from the full-text, rather than just the abstracts. One reason to suspect full-text classification outperformed abstract-only classification could be based on a reduced total number of features when considering the abstracts-only text. For instance, when considering the bag-of-words feature space, the imposed 80-instance threshold more than likely reduced the total number of potential features for classification using abstract text because unique phrases are less likely to occur frequently because of study and author specific terminology. To this point, the number of unique features used to classify all labels using abstracts text was 740, compared to 15,004 unique features using full article text. In addition, references are included as components of the full article text, so authors and article titles are also considered as features. References were included in the full-text assessments in part because of the demonstrated networks of author collaborations in the AuthorSynth tool (Sochat, 2015). Similarly, when considering the Cognitive Atlas feature space, terms may have not been represented as frequently (if at all) in the abstract text compared with the full article text. These findings are indicative of (1) more semantic variability across abstracts yielding fewer features with high enough frequency for classification purposes, and (2) less differentiation of features used for classification amongst labels, potentially leading to less accurate predictive performance.

### Bag-of-Words vs. Cognitive Atlas

Additionally, we sought to determine if a feature space derived from an expert defined vocabulary, the *Cognitive Atlas*, describing psychological constructs, mental operations, and experimental conditions could match or exceed the classification performance when using features derived from neuroimaging article text. This assessment was based on the premise that author-derived terms are non-specific with respect to the context of the article, and the frequency of terms associated with cognitive concepts and tasks from the *Cognitive Atlas* would be better suited for annotation using *CogPO* labels. These hypotheses are driven by evidence supporting dictionary matching algorithms in genetics research increasing prediction performance in concept recognition (Funk et al., 2016). When considering classification using full article text, the *bag-of-words* features outperformed the *Cognitive Atlas* features, though the difference (0.05) falls within the error range of consistency (0.20) of prediction accuracy for the *bag-of-words* approach. Additionally, if one considers the current scenario of article annotation using abstract text until full article text becomes more readily available, the *Cognitive Atlas* feature space actually outperforms *bag-of-words*. This finding, aside from gross feature representation differences in article abstracts (as reported above), supports the notion that article abstracts contain high-level, context specific terminology that *Cognitive Atlas* can leverage for classification purposes, whereas the *bag-of-words* features, which are subjected to a reduction technique that ensures sufficient power, show either (1) high semantic variability within a single label, or (2) low heterogeneity across all CogPO labels. Thus, while we generally identified comparable performance using the *Cognitive Atlas* feature space, we acknowledge that these findings are contextualized within the cognitive neuroimaging literature when using CogPO labels.

### Classification Algorithm

Based on overall performance, average Macro F1-scores across Cog PO labels and iterations were highest for the full-text *corpora* and bag-of-words *feature space* when using the logistic regression algorithm; although the performance was almost equivalent when using the support vector classifier algorithm. On average, the Bernoulli naïve Bayes and *k*-nearest neighbors algorithms failed to achieve equivalent predictive performance as the logistic regression and support vector classifiers, regardless of the *corpora* or *feature space* chosen. The Bernoulli naïve Bayes algorithm is based on binary feature representation; thus, frequency of appearance is not emphasized. The lack of emphasis on feature representation could be detrimental in weighting key terms used frequently about a specific cognitive domain, though it has been shown to be beneficial in document classification (McCallum and Nigam, 1998). The *k-nearest neighbors* algorithm annotates labels based on a majority vote of the *k* labels from the training-dataset with the smallest distance with the test-dataset. Annotation performance can thus vary based on the selected value of *k*, exhibits a U-shaped relationship with the number of relevant features (Okamoto and Yugami, 2003), and generally performs worse in the case of high-dimensional data (Mitchell et al., 1990). Aside from reduced performance levels, another limitation of the *k*-nearest neighbor algorithm is that it is computationally expensive regarding processing time and storage requirements, as no model is actually trained and distances must be calculated for every class. Support vector classifiers are robust and have been used for classification of cancer (Furey et al., 2000; Guyon et al., 2002), image (Chapelle et al., 1999) and audio (Guo and Li, 2003) classification, and identifying smokers compare to non-smokers (Pariyadath et al., 2014). In general, because of their ability to operate in high dimensional spaces, support vector classifiers have few drawbacks, with the exception of high processing times and memory consumption during the training and classification stages (Khan et al., 2010). Logistic regression is another of the more popular classification approaches for medical data classification (Dreiseitl and Ohno-Machado, 2002). Logistic regression models are generally less prone to overfitting and thus have a higher degree of generalizability. This is particularly important in the current context as there are unbalanced representations of CogPO labels used for training classifiers, and annotation of future articles may not be suspect to overfitting based on the data utilized in the current work.

### Feature Representation

Our exploratory analysis yielded word clouds for different clusters and demonstrated that anatomical terms appeared to dominate the most frequently utilized features for article classification across labels. This finding is important for two reasons: first, it suggests that semantic variability is greater for functional terms or task descriptors than anatomical labels; and second, frequently used anatomical terms are represented in a meaningful way that exhibit dense associations with similar cognitive concepts. For instance, it is not surprising to find that “superior temporal gyrus” is one of the most commonly utilized anatomical terms used to classify CogPO labels related to language (Friederici et al., 2003), or likewise the association between “amygdala” and emotion labels (Gallagher and Chiba, 1996). However, these anatomical terms are not domain-specific, and leveraging a feature space that weighs heavily toward anatomical descriptors could result in less confidence for article annotation, particularly in the cases where experimental designs are increasingly complex, interrogating multiple cognitive domains or brain networks. For instance, recent meta-analytic endeavors (Laird et al., 2015; Bottenhorn et al., 2018; Riedel et al., 2018) have demonstrated robust brain network activation across activation maps associated with distinct neuroimaging task paradigms. In this respect, a classification system whereby features are derived from an ontology of psychological concepts, such as the Cognitive Atlas, would rely more on authors’ discussion of experimental design and findings related to cognitive neuroscience and psychology. In this respect, efforts in text-mining the neuroimaging literature can be enhanced by referencing the genomics classification methodologies, as advanced concept and synonym recognition techniques are prevalent (Funk et al., 2016). Nonetheless, relationships between brain regions and neurological disorders can be delineated, providing invaluable knowledge of the either brain regions most commonly associated with specific disorders or, given the association between brain location with cognitive domains, which disorders are most commonly studied within a given domain. Finally, it is somewhat surprising that canonical brain networks did not emerge as frequently used features. Some of the most highly studied networks, such as the “default-mode” and “salience” networks reflect very little semantic variability. To this end, it would seem that authors tend to discuss their findings in terms of constituent components of these networks. Alternatively, the majority of the publications included in this assessment occurred prior to and including the year 2008, while seminal brain-network papers were published around that time (Seeley et al., 2007; Menon, 2011), indicating a lack of representation in the current database.

### Limitations and Future Directions

During the planning phase of our analyses, we considered the distinctions between CogPO and the Cognitive Atlas as developed ontologies for classification purposes. Ultimately, we believed that the Cognitive Atlas is more suitable to be leveraged as a feature space than as a label set because CogPO is meant to be more static, which fits the function of stable article annotations, whereas the Cognitive Atlas is meant to evolve. To this end, relationships between concepts in the Cognitive Atlas can be evaluated as weights between features for each classifier, and prediction performance can be improved as these relationships are further refined and Cognitive Atlas becomes more fully specified through crowd-sourcing efforts. Furthermore, evolving the Cognitive Atlas vocabulary to incorporate synonyms based on constituent parts of the features may serve to strengthen prediction performance (Funk et al., 2016).

Following best standards and practices, we only utilized CogPO labels that were annotated at least 80 times, which drastically reduced the number of labels used for classification. Thus, the context with which these results should be interpreted are with respect to those 86 labels that were trained and tested here. Public release will include classifiers for CogPO labels trained on the entire dataset. Additionally, as there were varying levels of performance across combinations of parameters, it is difficult to conclude that one combination is superior to the other. Using the full-text, bag-of-words, and logistic regression approach resulted in the best overall performance, but this was only slightly greater than when using the support vector machine classifier (and full-text, bag-of-words). Thus, subtle differences in classifier performance should be considered, and annotation performance in smaller datasets according to the classification algorithm should be investigated.

We utilized the largest known corpus of studies with manual annotations for deriving classifiers for CogPO labels, and as such, included all articles for training and testing purposes for labels to reach a sufficient power for analysis. An independent dataset is necessary for validation of the classifiers, and future work includes using manually annotated datasets to evaluate the ATHENA derived classifiers in the domain of executive function, social cognition, decision making, and cue reactivity. Furthermore, we are meta-analytically assessing whether spatial distinctions exist between executive control network depending on the specific nomenclature authors used to describe it (e.g., cognitive control network, executive function network, dorsal attention network, etc.).

All classifiers produced by the work performed may be integrated into existing tools, including Neurosynth, Brainspell and MetaCurious, and NiMARE. Neurosynth is a platform in which automated methods are used to extract relevant information from neuroimaging articles for the purpose of large-scale meta-analysis. These classifiers may be used to provide a new set of labels by which users can perform meta-analyses using Neurosynth’s database. Further development of the ATHENA classifiers through formal comparison with Neurosynth’s bag-of-words annotation approach is ongoing. Brainspell and MetaCurious allows researchers to search across the literature, manually curate collections of articles for meta-analyses, and add human annotation to the existing automated annotations for Neurosynth, which form the basis of the Brainspell database. The curation process involves adding labels to the articles, which can be used to improve ATHENA classifiers. Additionally, the classifiers may be used to improve the accuracy of targeted searches in MetaCurious, which will make comprehensive literature searches easier for meta-analysts. NiMARE is a Python package that implements a wide range of tools for neuroimaging meta-analysis, and it is in NiMARE that the ATHENA classifiers may be implemented and interact with Neurosynth and MetaCurious.

## Software Dependencies

As described above, the analyses presented in this work rely on the following dependencies: numpy (Van Der Walt et al., 2011), pandas (McKinney, 2011), statsmodels (Seabold and Perktold, 2010), SciPy (Jones et al., 2001), scikit-learn (Pedregosa et al., 2011), IPython (Pérez and Granger, 2007), nltk (Bird et al., 2009), pdfminer (Shinyama, 2007), seaborn (Waskom et al., 2017), and many core libraries provided with Python 2.7.11. Additionally, the ontological expansion of Cognitive Atlas term weights was influenced by Poldrack (2017)^14^.

## Author Contributions

AL, JT, and MT developed the study design. MR, TS, JH, and MT performed the coding and analyses. MR, TS, JH, MS, and AL contributed to the implementation. All authors contributed to the manuscript revision, and read and approved the submitted version.

## Conflict of Interest Statement

The authors declare that the research was conducted in the absence of any commercial or financial relationships that could be construed as a potential conflict of interest.

## Footnotes

1 pubmed.gov

2 neurosynth.org

3 brainmap.org

4 biopython.org

5 github.com/euske/pdfminer

6 cogpo.org

7 nltk.org

8 scikit-learn.org

9 github.com/NBCLab/athena

10 github.com/NBCLab/abbr

11 tysto.com/uk-us-spelling-list.html

12 cognitiveatlas.org

13 github.com/amueller/word_cloud

14 github.com/poldrack/cognitive_encoding_model/blob/master/neurosynth_prep/expand_ontology.py
