# Supplementary material for: Automated, Efficient, and Accelerated Knowledge Modeling of the Cognitive Neuroimaging Literature Using the ATHENA Toolkit
Source: Front Neurosci. 2019 May 15;13:494. doi: 10.3389/fnins.2019.00494 (PMC6530419; doi:10.3389/fnins.2019.00494)
Supplement: Supplementary file 1 [file Data_Sheet_1.docx]

**Supplemental Material**

**Supplemental Table 1. Cognitive Paradigm Ontology labels.** Labels for each CogPO dimension are listed, with an asterisk (*) following those labels that were included in the analysis. In total, there are 358 labels, and 86 labels remained after thresholding to only include those with at least 80 instances.

| **Behavioral Domain** | **Context** | **Diagnosis** | **Instruction** | **Paradigm Class** | **Response Modality** | **Response Type** | **Stimulus Modality** | **Stimulus Type** |
| --- | --- | --- | --- | --- | --- | --- | --- | --- |
| Action* | Aging | Alcoholism | Attend* | Acupuncture | Arm | Blink | Auditory* | AbstractGeometrical* |
| Action.Execution* | Disease* | Alexithymia | Count | AffectivePictures | Foot | BreathHold | Gustatory | Acupuncture |
| Action.Execution.Speech* | Emotion | Allodynia | Describe | AffectiveWords | Hand* | ButtonPress* | Interoceptive | AirPuff |
| Action.Imagination | ExperimentalDesign | AlzheimersDisease | Detect* | AntiSaccades | Leg | Draw | None* | AsianCharacters |
| Action.Inhibition* | Gender | AmyotrophicLateralSclerosis | Discriminate* | AutobiographicalRecall | None* | Drink | Olfactory | Braille |
| Action.MotorLearning | Genetic | AnorexiaNervosa | Encode* | BrailleReading | Ocular | FingerTapping | Other | Cartoons |
| Action.Observation | Handedness | AntisocialPersonalityDisorder | Fixate* | ChewingSwallowing | OralFacial* | FlexionExtension* | Tactile* | Clicks |
| Action.Preparation | Language | Anxiety | Generate* | ClassicalConditioning | Other | Grasp | Visual* | EMF |
| Action.Rest* | Learning | Aphasia | Identify | CountingCalculation | Pelvis | Manipulate |  | Electricity |
| Cognition* | NormalMapping* | AttentionDeficitHyperactivityDisorder | Imagine* | CuedExplicitRecognitionRecall* | Shoulder | Meditate |  | Faces* |
| Cognition.Attention* | NotDefined. | AutismSpectrumDisorders | Inhibit | CuedSpeech | Torso | Micturate |  | FacesandScenes |
| Cognition.Language* | Pharmacology* | BipolarDisorder* | Maintain | Deception | Wrist | None* |  | FalseFont |
| Cognition.Language.Orthography | Treatment | Blepharospasm | Meditate | DelayDiscounting |  | Point |  | FilmClip* |
| Cognition.Language.Phonology | | Blindness | Move* | DelayedMatchtoSample* | | Read |  | FixationPoint* |
| Cognition.Language.Semantics* | | BodyDysmorphicDisorder | Name | DividedAuditoryAttention | | Saccades |  | Food |
| Cognition.Language.Speech* | | BorderlinePersonalityDisorder | None | Drawing |  | Smile |  | Heat |
| Cognition.Language.Syntax | | BulimiaNervosa | PassiveRest* | Driving |  | Speech* |  | Infusion |
| Cognition.Memory* | | CerebralPalsy | Predict | EmotionInduction* |  | Swallow |  | Letters* |
| Cognition.Memory.Explicit* | | ChronicFatigueSyndrome | Read* | Encoding* |  | Whistle |  | Lettersandabstractobjects |
| Cognition.Memory.Implicit | | ChronicLowerBackPain | Recall* | EpisodicRecall |  | Write |  | MathematicalEquation |
| Cognition.Memory.Working* | | ConductDisorder | Repeat* | Estimation |  |  |  | Music |
| Cognition.Music |  | ConversionDisorder | Sing | FaceMonitorDiscrimination* | |  |  | Noise |
| Cognition.Reasoning* | | Deafness | Track | FigurativeLanguage | |  |  | None* |
| Cognition.SocialCognition* | | Dementia | Update | FilmViewing |  |  |  | Numbers* |
| Cognition.Soma |  | Depression* |  | FingerTappingButtonPress* | |  |  | Objects |
| Cognition.Space |  | DevelopmentalStuttering | | Fixation |  |  |  | Odor |
| Cognition.Time |  | Dyslexia |  | Flanker |  |  |  | Pain |
| Emotion* |  | EatingDisorderNotOtherwiseSpecified | | FlashingCheckerboard | |  |  | Pictures* |
| Emotion.Anger |  | EssentialBlepharospasm | | FlexionExtension |  |  |  | PlayingCards |
| Emotion.Anxiety |  | FetalAlcoholSyndrome | | FluencyInduction |  |  |  | Pseudowords |
| Emotion.Disgust |  | FibromyalgiaSyndrome | | Gambling |  |  |  | RandomDots |
| Emotion.Fear* |  | FragileXSyndrome | | GoNoGo* |  |  |  | ReversedSpeech |
| Emotion.Happiness* | | HuntingtonsDisease | | Grasping |  |  |  | Shapes* |
| Emotion.Happiness.Humor | | HypogeusiaorAgeusia | | HandEyeCoordination | |  |  | Sounds.Environmental |
| Emotion.Sadness |  | Hypothyroidism |  | HungerSatiety |  |  |  | Spinner |
| Interoception* |  | IntermittentExplosiveDisorder | | Hypercapnia |  |  |  | Syllables |
| Interoception.Bladder | | Lesions |  | ImaginedMovement | |  |  | Symbols* |
| Interoception.Hunger | | MeigesSyndrome |  | ImaginedObjectsScenes | |  |  | TMS |
| Interoception.RespirationRegulation | | MildCognitiveImpairment | | IsometricForce |  |  |  | TactileStimulation |
| Interoception.Sexuality | | MultipleSclerosis |  | LexicalDecision |  |  |  | Target |
| Interoception.Sleep | | Narcolepsy |  | MagnitudeComparison | |  |  | Tones* |
| Interoception.Thermoregulation | | NonschizophreniaPsychosis | | MagnitudeComparison.Distance | |  |  | Vibration |
| Interoception.Thirst | | Normals* |  | MagnitudeComparison.Luminance | |  |  | VideoGame |
| Perception* |  | Obesity |  | MagnitudeComparison.PhysicalSize | |  |  | Words* |
| Perception.Audition* | | ObsessiveCompulsiveDisorder | | MagnitudeComparison.SymbolicNumerical | | |  |  |
| Perception.Gustation | | Osteoarthritis |  | Meditation |  |  |  |  |
| Perception.Olfaction | | PanicDisorder |  | MentalRotation |  |  |  |  |
| Perception.Somesthesis* | | ParkinsonsDisease | | Micturition |  |  |  |  |
| Perception.Somesthesis.Pain* | | PathologicalGambling | | MotorLearning |  |  |  |  |
| Perception.Vision* |  | PostTraumaticStressDisorder | | MultiTasking |  |  |  |  |
| Perception.Vision.Color | | Psychopathy |  | MusicComprehension | |  |  |  |
| Perception.Vision.Motion* | | Psychosis |  | MusicProduction |  |  |  |  |
| Perception.Vision.Shape* | | PsychoticMajorDepression | | Naming |  |  |  |  |
|  |  | PureAutonomicFailure | | Naming.Covert |  |  |  |  |
|  |  | RecreationalDrugUsers | | Naming.Overt |  |  |  |  |
|  |  | RheumatoidArthritis | | ObjectManipulationDiscrimination | |  |  |  |
|  |  | Sadomasochism |  | OddballDiscrimination | |  |  |  |
|  |  | Schizophrenia* |  | OlfactoryMonitorDiscrimination | |  |  |  |
|  |  | SomatoformPainDisorder | | OrthographicDiscrimination | |  |  |  |
|  |  | Stroke |  | PainMonitorDiscrimination* | |  |  |  |
|  |  | SubstanceUseDisorder* | | PairedAssociateRecall | |  |  |  |
|  |  | Synesthesia |  | PassiveListening |  |  |  |  |
|  |  | TemporalLobeEpilepsy | | PassiveViewing* |  |  |  |  |
|  |  | TourettesSyndrome | | PhonologicalDiscrimination | |  |  |  |
|  |  | TraumaticBrainInjury | | PitchMonitorDiscrimination | |  |  |  |
|  |  | TraumaticSpinalCordInjury | | Pointing |  |  |  |  |
|  |  | Trichotillomania |  | Reading* |  |  |  |  |
|  |  | TurnerSyndrome |  | Reading.Covert |  |  |  |  |
|  |  | UnipolarDisorder |  | Reading.Overt |  |  |  |  |
|  |  | UrinaryIncontinence | | ReasoningProblemSolving | |  |  |  |
|  |  | VegetativeState |  | RecitationRepetition | |  |  |  |
|  |  | WritersCramp |  | RecitationRepetition.Covert | |  |  |  |
|  |  |  |  | RecitationRepetition.Overt | |  |  |  |
|  |  |  |  | Rest* |  |  |  |  |
|  |  |  |  | Reward* |  |  |  |  |
|  |  |  |  | Saccades |  |  |  |  |
|  |  |  |  | SelfReflection |  |  |  |  |
|  |  |  |  | SemanticMonitorDiscrimination* | |  |  |  |
|  |  |  |  | SequenceRecallLearning | |  |  |  |
|  |  |  |  | SexualArousalGratification | |  |  |  |
|  |  |  |  | Sleep |  |  |  |  |
|  |  |  |  | Stroop* |  |  |  |  |
|  |  |  |  | Stroop.ColorWord |  |  |  |  |
|  |  |  |  | Stroop.Counting |  |  |  |  |
|  |  |  |  | Stroop.Emotional |  |  |  |  |
|  |  |  |  | Stroop.Other |  |  |  |  |
|  |  |  |  | SyntacticDiscrimination | |  |  |  |
|  |  |  |  | TactileMonitorDiscrimination | |  |  |  |
|  |  |  |  | TaskSwitching |  |  |  |  |
|  |  |  |  | Taste |  |  |  |  |
|  |  |  |  | TheoryofMind |  |  |  |  |
|  |  |  |  | ThirstInduction |  |  |  |  |
|  |  |  |  | ToneMonitorDiscrimination | |  |  |  |
|  |  |  |  | TowerofLondon |  |  |  |  |
|  |  |  |  | TranscranialMagneticStimulation | |  |  |  |
|  |  |  |  | TraumaRecall |  |  |  |  |
|  |  |  |  | VibrotactileMonitorDiscrimination | |  |  |  |
|  |  |  |  | VideoGames |  |  |  |  |
|  |  |  |  | VisualMotion |  |  |  |  |
|  |  |  |  | VisualObjectIdentification | |  |  |  |
|  |  |  |  | VisualPursuitTracking | |  |  |  |
|  |  |  |  | VisuospatialAttention* | |  |  |  |
|  |  |  |  | Whistling |  |  |  |  |
|  |  |  |  | WisconsinCardSortingTest | |  |  |  |
|  |  |  |  | WordGeneration* |  |  |  |  |
|  |  |  |  | WordGeneration.Covert | |  |  |  |
|  |  |  |  | WordGeneration.Overt | |  |  |  |
|  |  |  |  | WordStemCompletion | |  |  |  |
|  |  |  |  | WordStemCompletion.Covert | |  |  |  |
|  |  |  |  | WordStemCompletion.Overt | |  |  |  |
|  |  |  |  | Writing |  |  |  |  |
|  |  |  |  | nback* |  |  |  |  |

| **Label** | **Count** |
| --- | --- |
| BehavioralDomain.Action | 127 |
| BehavioralDomain.Action.Execution | 72 |
| BehavioralDomain.Action.Inhibition | 56 |
| BehavioralDomain.Cognition | 839 |
| BehavioralDomain.Cognition.Attention | 206 |
| BehavioralDomain.Cognition.Language | 275 |
| BehavioralDomain.Cognition.Language.Semantics | 214 |
| BehavioralDomain.Cognition.Language.Speech | 110 |
| BehavioralDomain.Cognition.Memory | 216 |
| BehavioralDomain.Cognition.Memory.Explicit | 54 |
| BehavioralDomain.Cognition.Memory.Working | 171 |
| BehavioralDomain.Cognition.Reasoning | 46 |
| BehavioralDomain.Emotion | 373 |
| BehavioralDomain.Emotion.Disgust | 30 |
| BehavioralDomain.Emotion.Fear | 55 |
| BehavioralDomain.Emotion.Happiness | 45 |
| BehavioralDomain.Emotion.Sadness | 33 |
| BehavioralDomain.Perception | 254 |
| BehavioralDomain.Perception.Somesthesis | 93 |
| BehavioralDomain.Perception.Somesthesis.Pain | 91 |
| BehavioralDomain.Perception.Vision | 130 |
| BehavioralDomain.Perception.Vision.Shape | 79 |
| BehavioralDomain.Pharmacology | 60 |
| Context.Disease | 279 |
| Context.DiseaseEffects | 238 |
| Context.DrugEffects | 59 |
| Context.NormalMapping | 974 |
| Diagnosis.AutismSpectrumDisorders | 31 |
| Diagnosis.BipolarDisorder | 35 |
| Diagnosis.Depression | 33 |
| Diagnosis.Normals | 1106 |
| Diagnosis.Schizophrenia | 75 |
| Instruction.Attend | 300 |
| Instruction.Count | 33 |
| Instruction.Detect | 105 |
| Instruction.Discriminate | 607 |
| Instruction.Fixate | 137 |
| Instruction.Generate | 125 |
| Instruction.Move | 36 |
| Instruction.Name | 31 |
| Instruction.Passive/Rest | 135 |
| Instruction.Recall | 171 |
| Instruction.Repeat | 39 |
| OvertResponseModality.Hand | 766 |
| OvertResponseModality.None | 485 |
| OvertResponseModality.Oral/Facial | 96 |
| OvertResponseType.ButtonPress | 734 |
| OvertResponseType.None | 485 |
| OvertResponseType.Speech | 87 |
| ParadigmClass.Counting/Calculation | 36 |
| ParadigmClass.DelayedMatchtoSample | 45 |
| ParadigmClass.FaceMonitor/Discrimination | 172 |
| ParadigmClass.Go/No-Go | 45 |
| ParadigmClass.PainMonitor/Discrimination | 94 |
| ParadigmClass.PassiveViewing | 116 |
| ParadigmClass.Reward | 165 |
| ParadigmClass.SemanticMonitor/Discrimination | 172 |
| ParadigmClass.Stroop | 50 |
| ParadigmClass.Stroop.ColorWord | 30 |
| ParadigmClass.TaskSwitching | 35 |
| ParadigmClass.VisuospatialAttention | 38 |
| ParadigmClass.WordGeneration | 102 |
| ParadigmClass.WordGeneration.Covert | 61 |
| ParadigmClass.WordGeneration.Overt | 44 |
| ParadigmClass.n-back | 124 |
| StimulusModality.Auditory | 129 |
| StimulusModality.None | 132 |
| StimulusModality.Tactile | 84 |
| StimulusModality.Visual | 951 |
| StimulusType.Abstract/Geometrical | 50 |
| StimulusType.Digits | 97 |
| StimulusType.Faces | 157 |
| StimulusType.FixationPoint | 162 |
| StimulusType.Heat | 52 |
| StimulusType.Letters | 176 |
| StimulusType.None | 131 |
| StimulusType.Pictures | 251 |
| StimulusType.Pseudowords | 35 |
| StimulusType.Shapes | 199 |
| StimulusType.Symbols | 49 |
| StimulusType.Words | 384 |

**Supplemental Table 2. CogPO label frequency across articles.**  CogPO labels that were not assigned at least 80 times were not considered for further analysis on the basis of power, leaving a total of 86 labels remaining for testing classification performance across the variable parameters.

| **stop words** | | | | | | |
| --- | --- | --- | --- | --- | --- | --- |
| a | between | having | more | own | this | will |
| about | both | he | most | s | those | with |
| above | but | her | my | same | through | you |
| after | by | here | myself | she | to | your |
| again | can | hers | no | should | too | yours |
| against | did | herself | nor | so | under | yourself |
| all | do | him | not | some | until | yourselves |
| am | does | himself | now | such | up |  |
| an | doing | his | of | t | very |  |
| and | don | how | off | than | was |  |
| any | down | i | on | that | we |  |
| are | during | if | once | the | were |  |
| as | each | in | only | their | what |  |
| at | few | into | or | theirs | when |  |
| be | for | is | other | them | where |  |
| because | from | it | our | themselves | which |  |
| been | further | its | ours | then | while |  |
| before | had | itself | ourselves | there | who |  |
| being | has | just | out | these | whom |  |
| below | have | me | over | they | why |  |

**Supplemental Table 3. Stop words provided by the Natural Language Toolkit (NLTK).** Stop words are common transitional or descriptive terms that are not beneficial for classification purposes.

| **bnb** | |  | **svm** | | |  | **lr** | | |  | **knn** | | | |
| --- | --- | --- | --- | --- | --- | --- | --- | --- | --- | --- | --- | --- | --- | --- |
| alpha | frequency |  | C | gamma | frequency |  | C | penalty | frequency |  | n_neighbor | p | weight | frequency |
| 0.01 | 12.91 |  | 1 | 0.01 | 5.2 |  | 0.01 | l1 | 37.31 |  | 1 | 1 | distance | 0 |
| 0.1 | 3.75 |  | 1 | 0.1 | 1.66 |  | 0.01 | l2 | 0.34 |  | 1 | 1 | uniform | 2.25 |
| 1 | 22.05 |  | 1 | 1 | 26.77 |  | 0.1 | l1 | 5.17 |  | 1 | 2 | distance | 0 |
| 10 | 61.3 |  | 10 | 0.01 | 0.8 |  | 0.1 | l2 | 1.57 |  | 1 | 2 | uniform | 6.6 |
|  |  |  | 10 | 0.1 | 10.38 |  | 1 | l1 | 2.44 |  | 3 | 1 | distance | 0.23 |
|  |  |  | 10 | 1 | 37.15 |  | 1 | l2 | 6.26 |  | 3 | 1 | uniform | 2.25 |
|  |  |  | 100 | 0.01 | 3.86 |  | 10 | l1 | 2.76 |  | 3 | 2 | distance | 0.62 |
|  |  |  | 100 | 0.1 | 6.25 |  | 10 | l2 | 13.94 |  | 3 | 2 | uniform | 9.44 |
|  |  |  | 100 | 1 | 7.94 |  | 100 | l1 | 2.49 |  | 5 | 1 | distance | 0.66 |
|  |  |  |  |  |  |  | 100 | l2 | 27.72 |  | 5 | 1 | uniform | 3.91 |
|  |  |  |  |  |  |  |  |  |  |  | 5 | 2 | distance | 1.28 |
|  |  |  |  |  |  |  |  |  |  |  | 5 | 2 | uniform | 13.38 |
|  |  |  |  |  |  |  |  |  |  |  | 7 | 1 | distance | 0.71 |
|  |  |  |  |  |  |  |  |  |  |  | 7 | 1 | uniform | 4.43 |
|  |  |  |  |  |  |  |  |  |  |  | 7 | 2 | distance | 2.03 |
|  |  |  |  |  |  |  |  |  |  |  | 7 | 2 | uniform | 16.12 |
|  |  |  |  |  |  |  |  |  |  |  | 9 | 1 | distance | 1.04 |
|  |  |  |  |  |  |  |  |  |  |  | 9 | 1 | uniform | 5.47 |
|  |  |  |  |  |  |  |  |  |  |  | 9 | 2 | distance | 4.49 |
|  |  |  |  |  |  |  |  |  |  |  | 9 | 2 | uniform | 25.1 |

**Supplemental Table 4. *Hyperparameter* selection frequency.** The most frequently used combinations of *hyperparameters* for classifier tuning are expressed here as a percentage of the total number of iterations and folds across all labels (172,000).

| **Dimension** | **Behavioral Domain** | **Context** | **Diagnosis** | **Instruction** | **Paradigm Class** | **Response**  **Modality** | **Response**  **Type** | **Stimulus**  **Modality** | **Stimulus**  **Type** | **Overall** |
| --- | --- | --- | --- | --- | --- | --- | --- | --- | --- | --- |
| **Baseline** | | | | | | | | | | |
|  | 0.06 | 0.31 | 0.21 | 0.09 | 0.01 | 0.49 | 0.36 | 0.30 | 0.07 |  |
| overall performance | 0.52 (0.26) | 0.64 (0.33) | **0.69 (0.27)** | 0.31 (0.24) | 0.41 (0.27) | 0.59 (0.25) | 0.49 (0.29) | 0.60 (0.26) | 0.26 (0.24) |  |
| **Corpora** | | | | | | | | | | |
| abstract-only | 0.46 (0.27) | 0.59 (0.36) | 0.62 (0.32) | 0.26 (0.24) | 0.34 (0.28) | 0.53 (0.27) | 0.43 (0.30) | 0.56 (0.26) | 0.20 (0.22) | 0.39 (0.30) |
| full-text | **0.58 (0.23)** | **0.69 (0.30)** | **0.76 (0.17)** | **0.35 (0.24)** | **0.48 (0.25)** | **0.66 (0.21)** | **0.54 (0.28)** | **0.64 (0.26)** | **0.32 (0.25)** | **0.51 (0.27)** |
| **Feature Space** | | | | | | | | | | |
| bag-of-words | **0.52 (0.25)** | **0.69 (0.30)** | **0.69 (0.27)** | **0.34 (0.24)** | **0.42 (0.26)** | **0.63 (0.23)** | **0.51 (0.29)** | **0.63 (0.23)** | **0.32 (0.24)** | **0.47 (0.28)** |
| Cognitive Atlas | 0.52 (0.26) | 0.58 (0.35) | 0.69 (0.27) | 0.28 (0.24) | 0.40 (0.29) | 0.56 (0.27) | 0.46 (0.30) | 0.56 (0.29) | 0.20 (0.24) | 0.43 (0.30) |
| **Classifier** | | | | | | | | | | |
| Bernoulli naïve Bayes | 0.42 (0.27) | 0.60 (0.34) | 0.54 (0.25) | 0.24 (0.24) | 0.29 (0.24) | 0.54 (0.27) | 0.44 (0.31) | 0.52 (0.28) | 0.21 (0.22) | 0.36 (0.28) |
| k-nearest neighbor | 0.51 (0.22) | 0.58 (0.33) | 0.65 (0.26) | 0.30 (0.20) | 0.39 (0.23) | 0.59 (0.23) | 0.47 (0.28) | 0.59 (0.23) | 0.23 (0.19) | 0.43 (0.26) |
| logistic regression | 0.54 (0.29) | 0.64 (0.37) | 0.77 (0.26) | 0.26 (0.29) | 0.41 (0.33) | 0.59 (0.29) | 0.47 (0.33) | 0.60 (0.31) | 0.22 (0.29) | 0.45 (0.34) |
| support vector classifier | **0.62 (0.19)** | **0.72 (0.25)** | **0.80 (0.22)** | **0.43 (0.19)** | **0.54 (0.22)** | **0.67 (0.17)** | **0.57 (0.23)** | **0.69 (0.18)** | **0.38 (0.21)** | **0.56 (0.23)** |

**Supplemental Table 5. F1-scores for each variable.** Performance measures for each possible option from the potential variables *corpora*, *feature space*, and *classifier*, as indicated by average and standard deviations of the F1-scores across iterations for each Cognitive Paradigm Ontology dimension.

| **Dimension** | | **Behavioral Domain** | **Context** | | **Diagnosis** | **Instruction** | **Paradigm Class** | **Response Modality** | | **Response Type** | | **Stimulus Modality** | | **Stimulus Type** | | **Overall** |
| --- | --- | --- | --- | --- | --- | --- | --- | --- | --- | --- | --- | --- | --- | --- | --- | --- |
| **Baseline** | | | | | | | | | | | | | | | | |
|  | 0.06 | | 0.31 | 0.21 | | 0.09 | 0.01 | | 0.49 | | 0.36 | | 0.30 | | 0.07 |  |
| **Corpora + Feature Space** | | | | | | | | | | | | | | | | |
| **abstract-only** | |  |  | |  |  |  |  | |  | |  | |  | |  |
| bag-of-words | | 0.45 (0.28) | **0.63 (0.36)** | | **0.63 (0.33)** | 0.24 (0.24) | 0.33 (0.26) | **0.55 (0.26)** | | **0.43 (0.30)** | | **0.58 (0.25)** | | **0.24 (0.23)** | | **0.39 (0.30)** |
| Cognitive Atlas | | **0.47 (0.27)** | 0.55 (0.36) | | 0.60 (0.32) | **0.27 (0.23)** | **0.35 (0.29)** | 0.52 (0.29) | | 0.43 (0.30) | | 0.54 (0.27) | | 0.16 (0.19) | | 0.39 (0.30) |
| **full-text** | |  |  | |  |  |  |  | |  | |  | |  | |  |
| bag-of-words | | **0.60 (0.19)** | **0.75 (0.22)** | | 0.76 (0.18) | **0.43 (0.20)** | **0.52 (0.22)** | **0.70 (0.16)** | | **0.59 (0.26)** | | **0.69 (0.20)** | | **0.41 (0.21)** | | **0.55 (0.23)** |
| Cognitive Atlas | | 0.56 (0.25) | 0.62 (0.34) | | **0.77 (0.17)** | 0.30 (0.25) | 0.44 (0.28) | 0.61 (0.24) | | 0.50 (0.29) | | 0.58 (0.30) | | 0.23 (0.25) | | 0.47 (0.30) |
| **Corpora + Classifier** | | | | | | | | | | | | | | | | |
| **abstract-only** | |  |  | |  |  |  |  | |  | |  | |  | |  |
| Bernoulli naïve Bayes | | 0.38 (0.29) | 0.57 (0.36) | | 0.45 (0.29) | 0.22 (0.23) | 0.24 (0.24) | 0.52 (0.28) | | 0.40 (0.32) | | 0.51 (0.26) | | 0.18 (0.21) | | 0.32 (0.29) |
| k-nearest neighbor | | 0.48 (0.23) | 0.56 (0.37) | | 0.61 (0.31) | 0.27 (0.20) | 0.35 (0.24) | 0.53 (0.27) | | 0.43 (0.28) | | 0.56 (0.23) | | 0.20 (0.18) | | 0.40 (0.27) |
| logistic regression | | 0.43 (0.32) | 0.56 (0.40) | | 0.68 (0.34) | 0.16 (0.26) | 0.29 (0.33) | 0.48 (0.34) | | 0.39 (0.34) | | 0.53 (0.33) | | 0.11 (0.23) | | 0.34 (0.35) |
| support vector classifier | | **0.56 (0.21)** | **0.65 (0.29)** | | **0.72 (0.29)** | **0.37 (0.20)** | **0.47 (0.22)** | **0.60 (0.19)** | | **0.51 (0.24)** | | **0.64 (0.19)** | | **0.31 (0.18)** | | **0.50 (0.24)** |
| **full-text** | |  |  | |  |  |  |  | |  | |  | |  | |  |
| Bernoulli naïve Bayes | | 0.46 (0.24) | 0.63 (0.33) | | 0.62 (0.17) | 0.27 (0.24) | 0.34 (0.23) | 0.57 (0.28) | | 0.48 (0.29) | | 0.52 (0.30) | | 0.23 (0.22) | | 0.40 (0.27) |
| k-nearest neighbor | | 0.53 (0.21) | 0.61 (0.28) | | 0.70 (0.19) | 0.33 (0.19) | 0.43 (0.21) | 0.64 (0.18) | | 0.50 (0.28) | | 0.62 (0.23) | | 0.27 (0.18) | | 0.47 (0.24) |
| logistic regression | | 0.64 (0.22) | 0.71 (0.33) | | 0.86 (0.07) | 0.36 (0.28) | 0.54 (0.27) | 0.69 (0.19) | | 0.56 (0.30) | | 0.67 (0.28) | | 0.34 (0.30) | | 0.55 (0.30) |
| support vector classifier | | **0.68 (0.14)** | **0.80 (0.18)** | | **0.87 (0.08)** | **0.50 (0.16)** | **0.61 (0.19)** | **0.73 (0.12)** | | **0.63 (0.21)** | | **0.74 (0.15)** | | **0.45 (0.21)** | | **0.63 (0.20)** |
| **Feature Space + Classifier** | | | | | | | | | | | | | | | | |
| **bag-of-words** | |  |  | |  |  |  |  | |  | |  | |  | |  |
| Bernoulli naïve Bayes | | 0.46 (0.23) | 0.66 (0.28) | | 0.53 (0.23) | 0.34 (0.21) | 0.36 (0.21) | 0.61 (0.19) | | 0.50 (0.25) | | 0.59 (0.19) | | 0.33 (0.18) | | 0.43 (0.24) |
| k-nearest neighbor | | 0.46 (0.23) | 0.60 (0.32) | | 0.67 (0.26) | 0.26 (0.20) | 0.34 (0.23) | 0.59 (0.24) | | 0.46 (0.30) | | 0.58 (0.25) | | 0.23 (0.19) | | 0.40 (0.26) |
| logistic regression | | 0.56 (0.29) | 0.72 (0.33) | | 0.78 (0.27) | 0.32 (0.29) | 0.46 (0.32) | 0.62 (0.28) | | 0.50 (0.34) | | 0.66 (0.28) | | 0.33 (0.31) | | 0.50 (0.33) |
| support vector classifier | | **0.62 (0.20)** | **0.77 (0.24)** | | **0.80 (0.23)** | **0.42 (0.23)** | **0.53 (0.23)** | **0.69 (0.17)** | | **0.58 (0.24)** | | **0.71 (0.18)** | | **0.40 (0.24)** | | **0.57 (0.25)** |
| **Cognitive Atlas** | |  |  | |  |  |  |  | |  | |  | |  | |  |
| Bernoulli naïve Bayes | | 0.37 (0.30) | 0.54 (0.39) | | 0.55 (0.27) | 0.14 (0.22) | 0.22 (0.25) | 0.48 (0.33) | | 0.37 (0.34) | | 0.45 (0.33) | | 0.08 (0.18) | | 0.29 (0.31) |
| k-nearest neighbor | | 0.56 (0.20) | 0.57 (0.34) | | 0.64 (0.25) | 0.34 (0.19) | 0.44 (0.22) | 0.58 (0.22) | | 0.48 (0.26) | | 0.60 (0.22) | | 0.24 (0.19) | | 0.46 (0.25) |
| logistic regression | | 0.51 (0.30) | 0.55 (0.39) | | 0.76 (0.26 | 0.20 (0.27) | 0.37 (0.34) | 0.55 (0.30) | | 0.45 (0.32) | | 0.54 (0.34) | | 0.11 (0.23) | | 0.40 (0.35) |
| support vector classifier | | **0.62 (0.17)** | **0.68 (0.26)** | | **0.79 (0.21)** | **0.45 (0.15)** | **0.55 (0.20)** | **0.65 (0.18)** | | **0.56 (0.22)** | | **0.67 (0.18)** | | **0.36 (0.18)** | | **0.56 (0.22)** |

**Supplemental Table 6. F1-scores for pairs of variables.** Performance measures for each possible combination of options from the potential variable pairings, as indicated by average and standard deviations of the F1-scores across iterations for each Cognitive Paradigm Ontology dimension.


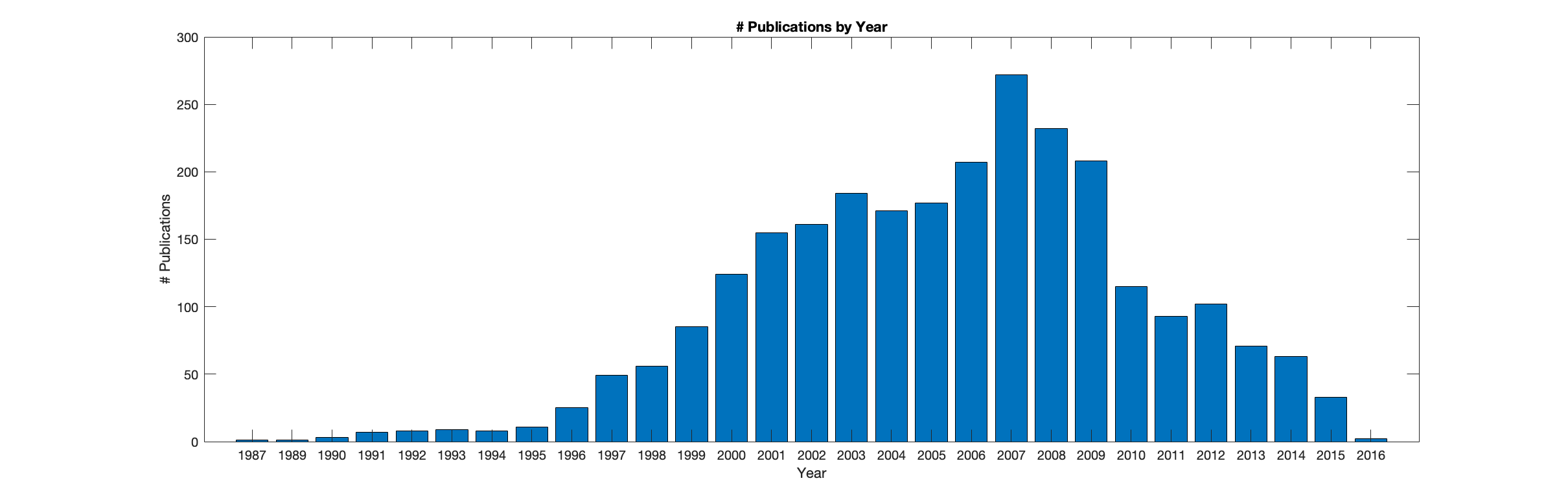


**Supplemental Figure 1. Publications by Year in BrainMap database.** The number of publications by year archived in the BrainMap database. The peak number of neuroimaging articles archived in BrainMap by year occurred in 2008. This is according to publication year, not when the article was archived in BrainMap.


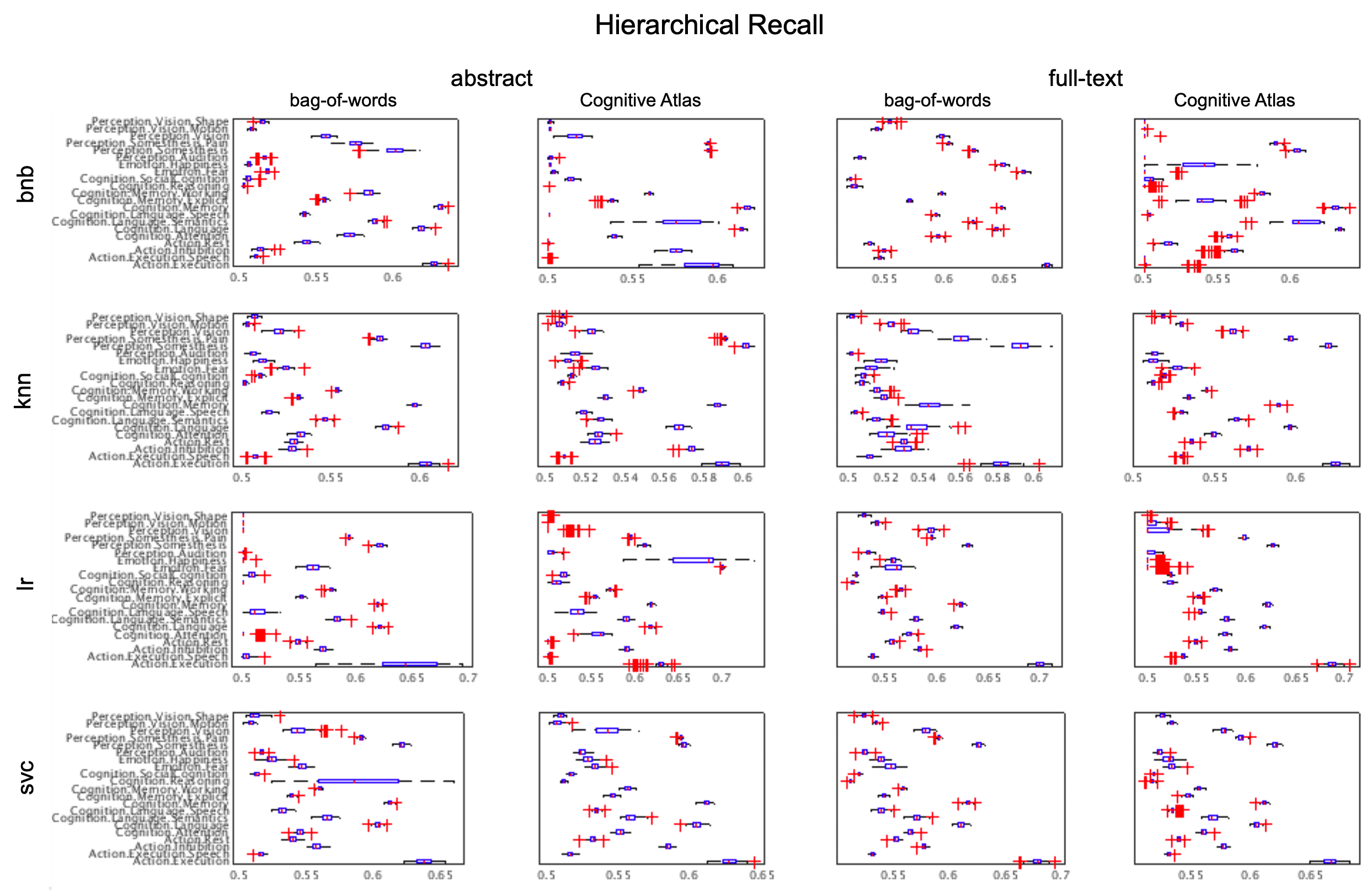


**Supplemental Figure 2. Hierarchical recall distributions.** Hierarchical recall distributions were calculated for each label across iterations for each combination of corpora, feature space, and classifier.


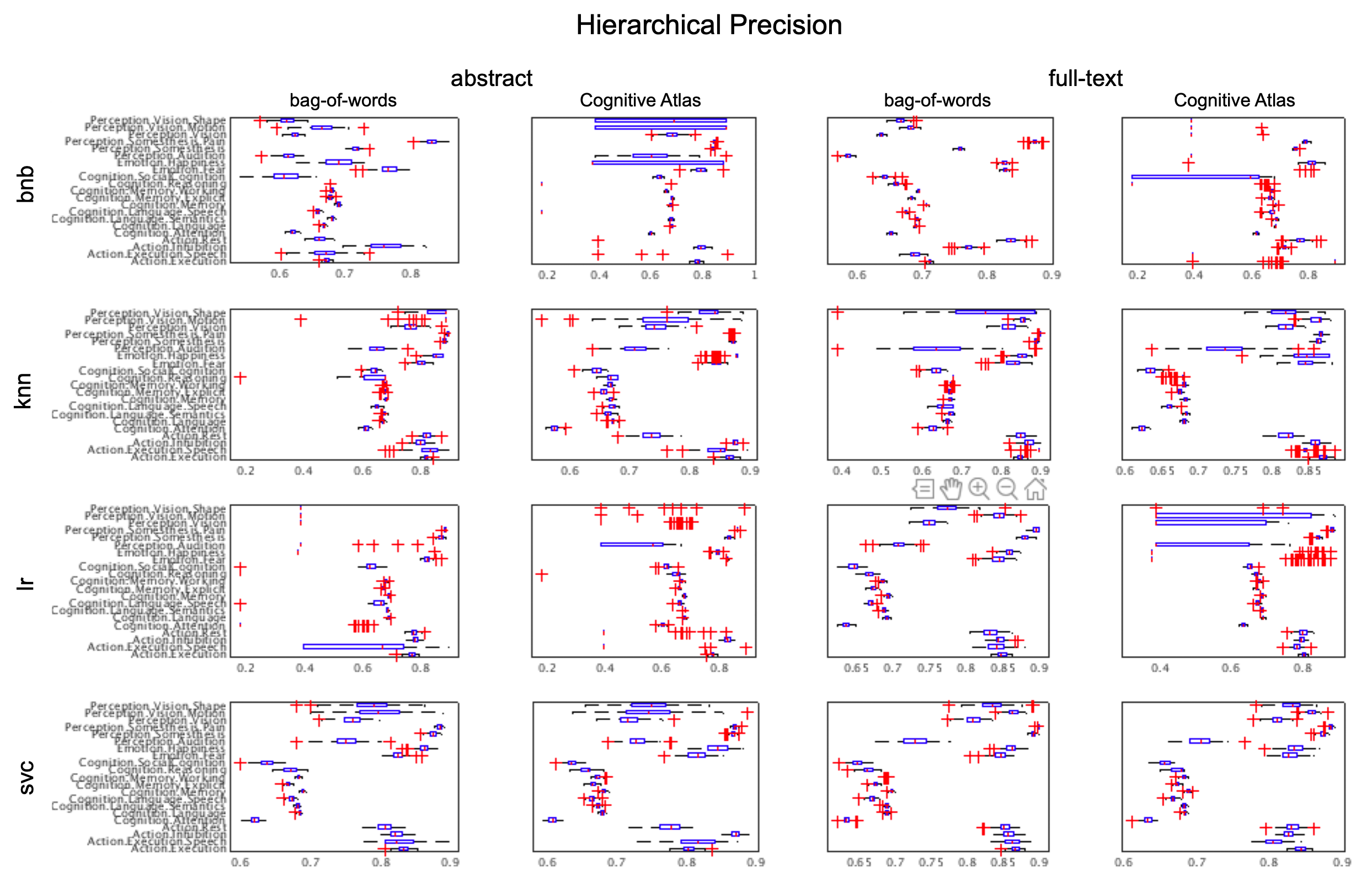


**Supplemental Figure 3. Hierarchical precision distributions.** Hierarchical precision distributions were calculated for each label across iterations for each combination of corpora, feature space, and classifier.

**
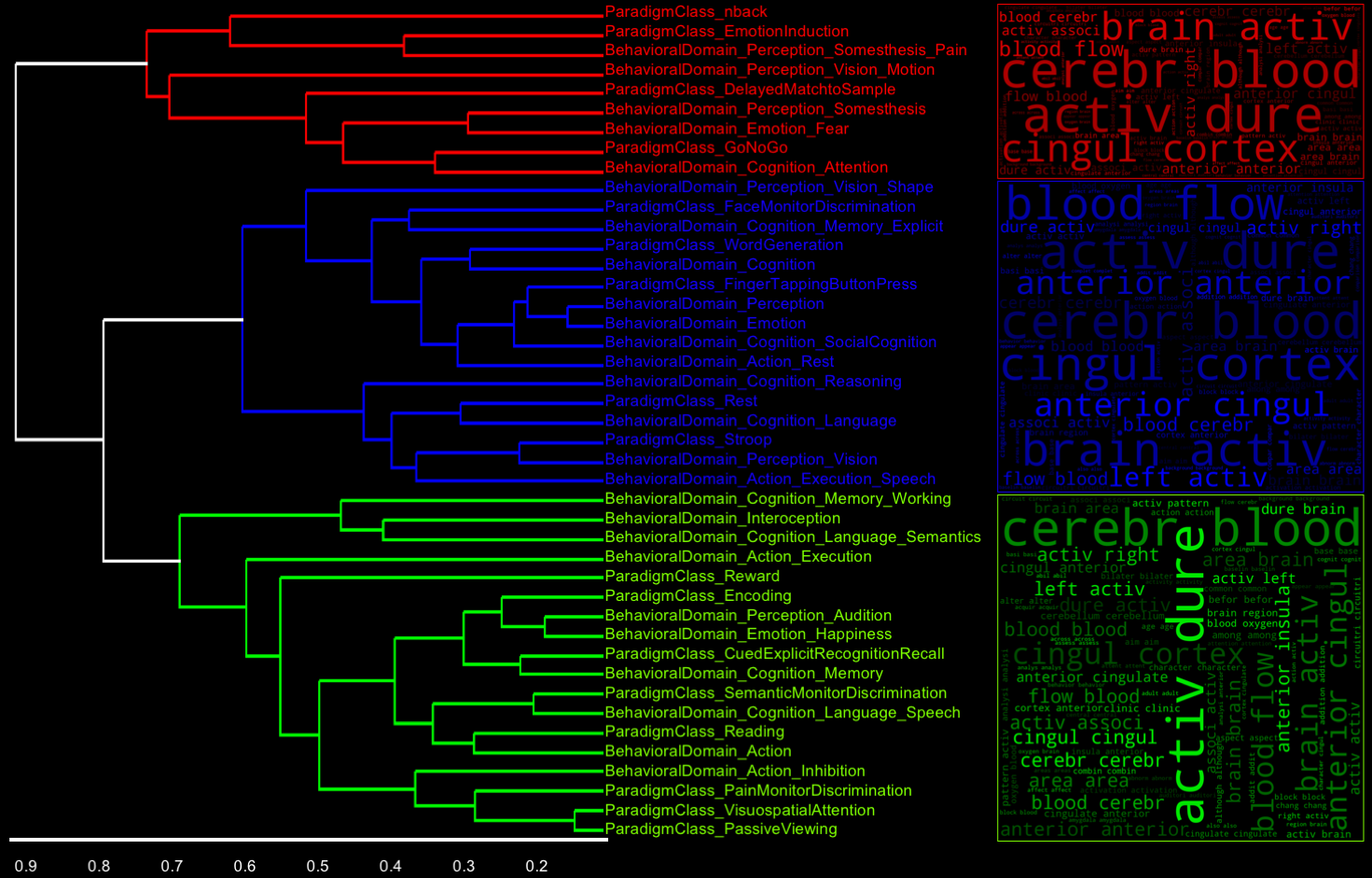
**

**Supplemental Figure 4.** **Label similarity dendrogram using features from abstracts.** Similarity between Behavioral Domain and Paradigm Class metadata labels based on features selected for classification across folds and iterations using article abstract text. Clusters are representative of labels and their corresponding manuscript in which similar language was used within the abstracts.

**Supplemental Text**

***tf-idf* Vectorization.**

*tf* is the term-frequency and *tf-idf* is the term-frequency times inverse document-frequency, defined as:

$$tf-idf\left( t,d \right)=tf(t,d)\times idf(t)$$

where *t* is the term and *d* is the document. Thus, *tf(t,d)* corresponds to the frequency of a term within a document and *idf(t*) is the inverse document frequency for a given term, calculated as*:*

$$idf\left( t \right)=log\left( \frac{1+n}{1+df(t)} \right)+1$$

where *n* is the total number of documents in the document set and *df(t)* is the number of documents in the document set that contain term t. This information can be found in more detail at https://scikit-learn.org/stable/modules/feature_extraction.html#tfidf-term-weighting.

**Radial Basis Kernel Function**

$$K\left( \boldsymbol{x},\boldsymbol{x'} \right)=exp\left( -\frac{\left\| \boldsymbol{x}-\boldsymbol{x'} \right\|^{2}}{2\sigma^{2}} \right)$$

where ||**x**-**x’**||^2^ is the squared Euclidean distance between the two feature vectors and σ is a free parameter. The parameter σ determines the extent to which a single article in the training-dataset influences the classifier.

**F1-Score Derivation.**

The F1-score is defined as the harmonic mean of precision and recall, where:

$$F1=2\times\frac{precision\times recall}{precision+recall}$$

$$precision=\frac{tp}{tp+fp}$$

$$recall=\frac{tp}{tp+fn}$$

Here, *tp* stands for true-positives, the number of articles that were predicted to have the correctly annotated label, *fp* stands for false-positives, the number of articles that were predicted to have a given label of interest, but they were actually not annotated with that label, and *fn* stands for false-negatives, the number of articles that were not predicted to have the label of interest but were actually annotated with the label.
